# A high-throughput screening platform to facilitate treatment development in Rett syndrome

**DOI:** 10.3389/fneur.2026.1759410

**Published:** 2026-05-08

**Authors:** Yanhong Yin, Anxin Wang, Qiping Dong, Ziyao Zhang, Qian Bu, Runfeng Wang, Qiang Chang

**Affiliations:** Waisman Center, University of Wisconsin-Madison, Madison, WI, United States

**Keywords:** high-throughput screening (HTS), human embryonic stem cells (hESCs)/induced pluripotent stem cells (iPSCs) derived neurons/astrocytes, isradipine, JC-10-based mitochondrial membrane potential (MMP) assay, leucine rich repeating containing 17 (*LRRC17*), methyl-CpG-binding protein 2 (*MECP2*), Rett syndrome (RTT)

## Abstract

Rett syndrome (RTT) is a rare X-linked progressive neurodevelopmental disorder affecting predominantly females with no cure and a prevalence of ~1 in 10,000 female birth worldwide. Before mutations in the methyl-CpG binding protein 2 (*MECP2*) gene were identified to cause classic RTT, there were suggestions that RTT is a mitochondrial disease. Being an essential organelle for all eukaryotic cells, the mitochondria produce energy, buffer calcium, and regulate the generation of reactive oxygen species. Indeed, accumulated reports documented mitochondrial abnormalities in RTT patient biopsies, and animal models and human stem cell models of RTT, including reduced ATP production, altered mitochondrial structure, increased systemic oxidative stress, abnormal calcium activity, mtDNA copy number, and deficiencies in mitochondrial enzyme activity. While it remains unclear how loss of MECP2 function leads to wide-ranging mitochondrial deficits, improving mitochondrial function could still bring benefits to RTT patients. After defining the mitochondrial membrane potential deficit in astrocytes differentiated from RTT patient-specific induced pluripotent stem cells (iPSC), we established a novel high-throughput screening (HTS) platform based on the JC-10 mitochondrial membrane potential (MMP) assay, which served as a rapid primary readout. All primary hits were subsequently validated by independent functional assays to confirm their effects on mitochondrial health. Using this system, we performed a small-molecule screening of 1,134 selected US Food and Drug Administration (FDA)-approved drugs and a small interfering RNA (siRNA) screening of 336 genes upregulated in RTT astrocytes and identified candidate drugs and candidate genes that reversed the MMP deficits in RTT astrocytes. Among the candidate drug hits, isradipine, a dihydropyridine calcium-channel blocker, provided preliminary evidence of neuroprotective effects both *in vitro* and *in vivo*. Among the candidate gene hits, *LRRC17*, a gene encoding a secreted protein, emerged as a strong candidate mediator whose elevated levels are strongly associated with and likely contribute to various observed cellular deficits. siRNA knockdown of *LRRC17* not only rescued mitochondrial dysfunction in RTT astrocytes but also reversed deficits in neurons cultured in astrocyte-conditioned media. Our study provides new insights into mitochondrial dysfunction in RTT and establishes an HTS platform for the initial identification of novel therapeutic targets for follow-up studies.

## Introduction

Rett syndrome (RTT) is a rare X-linked progressive neurodevelopmental disorder that predominantly affects females with no cure available to date. Its global prevalence is approximately 1 in 10,000 female births. Prior to the discovery that mutations in the methyl-CpG binding protein 2 (*MECP2*) gene underlie 95% of the classic RTT cases (1), RTT was hypothesized to be a mitochondrial disease (2). As a vital organelle in all eukaryotic cells, the mitochondrion fulfills multiple essential functions, including energy production. In RTT, much evidence indicates that these core mitochondrial processes are profoundly dysregulated, manifesting as altered mitochondrial morphology, elevated systemic oxidative stress, perturbed mtDNA copy number, and deficiencies in mitochondrial enzyme activity (3–10). Early transcriptomic analyses in mouse models of Rett syndrome identified widespread dysregulation of mitochondrial gene expression in astrocytes, establishing a link between astrocyte mitochondrial dysfunction and disease pathophysiology (11). Recent multi-omics analyses have further defined the molecular signatures and pathway dysregulations underlying these abnormalities, highlighting the intricate links between MECP2 loss-of-function and mitochondrial impairment in RTT pathogenesis (12). Although the pathogenic mechanisms linking *MECP2* mutations to the diverse clinical manifestations of RTT remain incompletely elucidated, emerging studies suggest that RTT pathogenesis involves a complex interplay of redox imbalance and inflammation (i.e., oxinflammation), impaired mitophagy, synaptic and metabolic dysfunction, proteasomal impairment, unfolded protein response (UPR) activation, and mechanistic target of rapamycin (mTOR) pathway dysregulation, with mitochondrial abnormalities proposed to play a central role in coordinating these pathological processes (13–19). Notably, mitochondrial modulation strategies, such as leriglitazone administration, have shown preliminary therapeutic potential in preclinical models, underscoring the validity of targeting mitochondrial function for RTT treatment (20). Not surprisingly, recent advances in AI-aided mitochondrial phenotyping have further supported the feasibility of high-throughput screening for mitochondrial modulators in neurodevelopmental disorders (21).

Since RTT is a neurological condition and MECP2 expression is the highest in neurons, the vast majority of RTT research has naturally focused on neurons (22–29). Yet studies on astrocytes in the past decade have clearly demonstrated that loss of MECP2 in astrocytes causes neuronal defects and restoring MECP2 expression in astrocytes alleviates disease symptoms (30–35). Recent work has further refined our understanding of astrocyte-mediated pathogenesis in RTT: *Mecp2* knockout astrocytes were shown to disrupt neuronal synaptogenesis via interleukin-6 (IL-6)-dependent mechanisms (36), while *MECP2* mutations were found to severely impair key cellular and molecular signatures during human astrocyte maturation (34). Critically, mitochondrial dysfunction and enhanced ROS production in *MECP2* mutant astrocytes have been identified as potential drivers of non-cell-autonomous neuronal damage (35), with dysregulation of the glutamine transporter SNAT1 further implicated in mediating astrocyte mitochondrial dysfunction and neurotoxicity in RTT (37), suggesting astrocyte mitochondria as a potential therapeutic target.

The development of human induced pluripotent stem cell (iPSC) models has enabled more mechanistic investigations into RTT pathophysiology, overcoming some limitations of purely murine systems (38). Work in our lab first established astrocytes differentiated from human embryonic stem cell (hESC) lines carrying *MECP2* deletion (*MECP2^KO^*) or RTT disease causing mutations (*MECP2^T158M^*) and RTT patient-specific induced pluripotent stem cell (iPSC) lines (*MECP2^R294X^*, *MECP2^v247fs^*) and their corresponding isogenic controls and characterized both cell-autonomous and non-cell-autonomous phenotypes (31). Recently, using the JC-10 assay to quantify mitochondrial membrane potential (MMP), we observed a significantly reduced ratio of red/green (F590/F520) fluorescence in neurons differentiated from hESCs expressing mutant *MECP2* or no *MECP2* compared with the wild-type neurons (24), indicating a less polarized MMP (a sign of less healthy mitochondria) in *MECP2* mutant neurons. Most recently, we have observed the same MMP deficit in RTT astrocytes.

Recognizing that the MMP phenotype can be quickly and reliably assessed and quantified in live cells using the JC-10 assay, we reasoned it could serve as a rapid primary readout for HTS to develop treatments targeting mitochondrial dysfunction in Rett syndrome, with subsequent validation required to confirm effects on overall mitochondrial function. To explore the feasibility of such a platform, we designed and performed two complementary screenings. The first was a small molecule screening of a library of 1,134 FDA–approved drugs, and the second was an siRNA library targeting 336 genes upregulated in RTT astrocytes. We identified candidate drugs and candidate genes that reversed the MMP deficits in RTT astrocytes in our primary screening, and validated those hits in multiple lines of RTT astrocytes for both primary MMP readout and additional mitochondrial function assays to confirm their effects on mitochondrial function beyond MMP. Among the candidate drug hits, isradipine, a dihydropyridine calcium-channel blocker, exhibited neuroprotective effects both *in vitro* (in RTT hESCs/iPSCs-derived astrocytes and neurons) and *in vivo* (in a RTT mouse model). Among the candidate gene hits, *LRRC17* (Leucine Rich Repeat Containing 17), which encodes a secreted protein, was detected at higher level in RTT astrocytes and medium conditioned by RTT astrocytes. Knocking down LRRC17 using siRNA in RTT astrocytes not only rescued mitochondrial dysfunctions (including improved MMP, ATP production, and mitochondrial morphology) in RTT astrocytes but also reversed neuronal deficits in neuron-astrocyte co-cultures. Thus, our study provides novel insights into mitochondrial dysfunctions in RTT and establishes a high-throughput screening platform for initial identification of novel therapeutic targets.

## Materials and methods

### Animal research

All animal procedures were performed according to protocols approved by the Institutional Animal Care and Use Committee at the University of Wisconsin-Madison. Mice were randomized into the treatment/control groups at 5 weeks of age using a computer-generated randomization list, ensuring balanced group sizes. All symptoms were scored by an experimenter blinded to the treatment group allocation. Mice were euthanized if they exhibited >20% body weight loss, hindlimb paralysis, or inability to access food/water, in accordance with IACUC guidelines. Only male *Mecp2*^−/y^ mice were used, which carry a targeted deletion of exons 3, resulting in the loss of full-length MeCP2 protein expression. This truncated version lacks the methyl-DNA binding domain and is not functional (39).

### Cell lines

This study used congenic pairs of wild-type (WT) and mutant (MT) Rett syndrome patient-specific induced pluripotent stem cell (iPSC) lines carrying the R294X and the V247fs mutations, as well as human embryonic stem cell (hESC) line H9 carrying the T158M mutation and *MECP2* knockout (KO) cell lines. Wild-type hESC line H9 was obtained from WiCell Research Institute (RRID:SCR_004364; www.wicell.org). Isogenic gene-corrected WT control iPSC lines were generated from the patient-derived mutant iPSCs via CRISPR/Cas9-mediated genome editing (24, 31). All cell lines were routinely tested to ensure correct genotype identification as described in our lab’s previous studies (24, 31). The H9-GFP reporter hESC line was generated by genetic modification and kindly provided by Dr. Suchun Zhang’s lab. All parental hESC and iPSC lines were quality-verified by WiCell Research Institute with official QC reports confirming correct karyotypes and no contamination. All astrocytes and neurons used in all experiments were derived from these validated cell stocks and tested negative for contaminations. A detailed summary of cell line usage across all experiments is provided in Supplementary Table S1.

### Neuron and astrocyte differentiation

Human forebrain neurons were differentiated from hESCs/iPSCs as previously described (40, 41). Human astrocyte differentiation was performed as previously described with minor modification (42). Briefly, ESCs were used at passages 27–39 and iPSCs were used at passages 7–15. ESCs/iPSCs were induced to neural progenitors (NP) which were freshly generated within 1 month, without additional passaging, and directly differentiated into mature neurons thereafter. For astrocyte differentiation, astrocyte progenitors (AP) were selected at a mature stage of 8–12 months (with a minimum maturation period of 6 months) for subsequent terminal differentiation into astrocytes. All astrocytes and neurons were freshly differentiated) from NP/AP cells; for each genotype, at least 3 independent differentiation batches were used for drug screening, 2 for siRNA screening, and 6 for all functional validation assays.

### Screenings

Selected drugs were used at a concentration of 1 μM and incubated for 4 h. siRNA pool (including 4 different siRNA against each target gene, Dharmacon) of target genes were added to freshly differentiated astrocytes after 7 days, and incubated for 3 days followed by the JC-10 assay in Matrigel-coated 96-well plates (CellCarrier-96 black, Perkin-Elmer) at an optimized density of 1.5 × 10^4^ cells per well. Cells were uniformly seeded across all wells of each plate using a mechanical multi-channel dispensing instrument from a single homogenous cell suspension, ensuring no inter-well cell density variation; no further cell number normalization was needed. For drug screening, each plate contained 3 wells of untreated R294X wild type (WT) astrocytes as positive controls, 3 wells of untreated R294X mutant (MT) astrocytes, and 2 wells of R294X MT astrocytes treated with 1 μM FCCP as negative controls, which were selected based on the manufacturer’s protocol to confirm the assay operated within its linear response range for mitochondrial membrane potential (MMP) measurements. The mean MMP fluorescence ratio of each well, calculated from the average signal intensity of the entire well, was normalized against that of the untreated WT. Assay reliability for drug screening was validated by Z′-factor calculation for each plate, with values ranging from 0.09 to 0.51 across all plates; intra-assay duplicate reproducibility was assessed by coefficient of variation (CV), with all samples exhibiting CV < 15% (mean 8.5%, median 7.6, and 83.2% of samples had CV < 10%). The global mean and SD across all plates were calculated as 0.687 and 0.173. The SD-based thresholds are applied in a distribution-appropriate manner. The drug distribution is clearly bimodal, indicating two biologically distinct populations corresponding to non-responders and candidate rescuing compounds. Under such conditions, SD-based thresholds computed from the pooled distribution are inappropriate because the variance is inflated by mixing populations. We therefore modeled the drug screening using a two-component Gaussian mixture model, identifying a dominant high-response component (mean = 0.734; SD = 0.085; weight ≈ 0.91) and a low-response component. Drug hits were defined as compounds with F590/F520 values ≥2 SD above the mean of the high-response component (threshold = 0.903). This criterion yields 20 drug candidates, providing a principled, distribution-aware alternative to an arbitrary pooled SD cutoff. Formal multiple-testing correction (e.g., FDR) was not applied at the primary screening stage, consistent with standard practice. Instead, false-positive elimination relied on independent biological validation. All primary hits from the drug screening were further validated by repeating the JC-10 assay using Confocal A1 microscope (to confirm the primary JC-10 results from the Operetta system), alongside secondary assays including ATP level and mitochondrial morphology analysis, ensuring functional relevance of positive findings independent of the initial screening cutoff. No compounds were excluded or repeated for poor signal quality, and no plates were discarded for compound-induced toxicity in drug screening. A detailed summary of validation assays performed for each drug hit is provided in Supplementary Table S2.

For siRNA screening, the 336 upregulated genes in R294X astrocytes were derived from our unpublished RNA-sequencing dataset. Detailed gene information is provided in Supplementary Table S3. Each plate contained internal plate control wells: 4 wells of WT astrocytes as positive controls, 4 wells of untreated MT astrocytes as negative controls and 80 wells of MT astrocytes treated with 5 pM/1 μL siRNA pool premixed with 0.6 μL Lipofectamine RNAiMAX Transfection Reagent (Invitrogen, Catalog: 13778500). Non-targeting siRNA (siGLO control) served as a control for transfection efficiency and successful siRNA delivery. The mean MMP fluorescence ratio of siRNA screening controls in each plate, calculated from the average signal intensity of the entire well, was set with WT as the reference (1.00 ± 0.09). Assay reliability for siRNA screening was validated by Z′-factor calculation for each plate, with values ranging from 0.302 to 0.370 across all plates. Duplicate well CV analysis confirmed consistent intra-assay performance, aligning with the same CV acceptability criteria (CV < 15%) applied for drug screening. The siRNA distribution is approximately unimodal with a right-skewed tail. Under this structure, a standard deviation–based threshold is appropriate. A ≥ 2 SD cutoff captures rare high-effect genes while retaining biologically validated candidates, whereas more stringent cutoffs (≥3 SD) exclude confirmed hits. We therefore retain ≥2 SD as the siRNA hit criterion (global mean = 0.710; global SD = 0.084; ≥2 SD threshold = 0.879). Using this hit criterion, we identified 9 candidates. FDR was not applied at the primary screening stage, consistent with standard practice. Instead, false-positive elimination relied on independent biological validation. All primary siRNA hits identified via Operetta-based high-throughput screening were subsequently re-validated using A1 Confocal microscopy for JC-10 assay (to confirm MMP results) using multiple *MECP2* mutation cell lines and functional relevance assessment, including ATP detection and mitochondrial morphology and calcium activity analysis, ensuring our findings extend beyond SD-based cutoffs. No siRNA-treated samples were excluded for poor signal or toxicity. A detailed summary of validation assays performed for each siRNA hit is provided in Supplementary Table S4.

### JC-10 assay

MMP was determined by the JC-10 Assay Kit (Abcam, Catalog#ab112134), following the manufacturer’s protocol. Astrocytes were imaged using the PerkinElmer Operetta^®^ or a Nikon A1RSi confocal microscope system with a 20×, 60×, or 100× objective. The MMP was determined by the ratio of the intensity of red fluorescence (emission wavelength 590 nm) to green fluorescence (emission wavelength 520 nm). For confirmatory confocal imaging, 6 representative fields of view were captured per condition. Individual cell bodies were segmented in ImageJ via thresholding, and the red/green ratio was calculated per analyzed cell, with the mean value of each acquired image used for statistical analysis.

### Measurement of cellular ATP

Levels of cellular ATP were quantified using the ATPlite^™^ Luminescence ATP Detection Assay (Perkin-Elmer) according to the manufacturer’s instructions. The luminescence was measured using a GloMax Multi + platereader (Promega). The concentration of ATP was calculated with the calibration curves which were constructed at six calibration standards samples. All measurements were normalized to cell numbers to account for variations in cellular density across samples, as described in detail in the Results section.

### Neuronal morphology

Neurons differentiated from H9-GFP hESCs (H9 hESCs constitutively expressing GFP) were co-cultured with conditioned RTT iPSCs derived astrocytes or astrocyte media for 3 days and were imaged on an A1RSi confocal microscope (Nikon) with a 20×, 60×, or 100× objectives. Z-stack (at 1 μm intervals) images (1,024 × 1,024 pixels) were acquired using a 100× oil objective. Semi-automated image analysis was performed using Bitplane IMARIS 8.0. 3D image analysis software (Oxford Instruments, Concord, MA). Data was extracted for Sholl analysis to quantify total dendritic length and terminal point number of each GFP-positive neuron.

### Mitochondrial morphology

Mitochondrial morphology was assessed using Rh123 (Thermo Fisher Scientific, catalog # R302) staining for 30 min at 37 °C for drug validation. TOMM20 immunostaining was used to assess mitochondrial morphology for siRNA validation. Nuclei were stained with DAPI for 5 min before imaging. The aspect ratio (AR) and form factor (FF) of mitochondria were calculated using ImageJ (RRID:SCR_003070) with mitochondrial morphology plug-in as previously described (43). To minimize potential bias in these assessments, all staining procedures were standardized across groups, and image analysis was performed in a blinded manner using automated ImageJ software, as detailed in the Materials and Methods.

### *In vitro* calcium imaging

As previously described (40), all image data were acquired in frame-scanning mode at a rate of 1 frame every 2 s. Fluo-4 was excited at 488 nm. The Ca^2+^ imaging data were analyzed using custom-written programs in Python. The metadata and image data of the raw images were read with python-bioformats. Spontaneous Ca^2+^ elevations from the soma and processes of astrocytes were analyzed separately. For each coverslip, a minimum of 6 distinct fields of view were imaged to ensure adequate and representative sampling of the astrocyte population. Ca^2+^ amplitude and frequency were quantified at the single-cell level, with each cell treated as one independent observation. Importantly, cells were obtained from at least three independent differentiations per group, and differentiations were performed independently on separate days to ensure experimental reproducibility.

### ELISA assay

Astrocyte culture media were collected and assayed for LRRC17 protein with an ELISA kit (Biomatik, catalog # EKC34406) according to manufacturer instructions. All samples from a given batch were assayed within the same assay plate. The detection range is 23.44 pg/mL–1,500 pg/mL, and sensitivity of the assay (stated by the manufacturer) was 5.86 pg/mL. The concentration of LRRC17 protein was calculated with standard curve.

### Western blot

Cultured astrocytes from 6-well plate were lysed in ice-cold RIPA lysis buffer, containing protease inhibitor mixture (Roche), loading into 10% SDS-PAGE gel. For immunodetection, the following antibodies were used: rabbit anti-LRRC17 (Novus catalog# NBP1-83309, RRID:AB_11001463, 1:800; Proteintech catalog # 20918-1-AP, 1:300); anti-GAPDH (Millipore catalog # AB2302 RRID:AB_10615768). After incubation with DyLight dye-conjugated secondary antibodies (Thermo Fisher Scientific catalog #35518 RRID:AB_614942; #SA5–35571 RRID:AB_2556775; 1:10,000) for 1 h at room temperature, blots were scanned using the Odyssey Western Detection system (LI-COR Biosciences), followed by quantification with ImageStudio software (LI-COR Biosciences).

### Immunostaining

The immunostaining experiments were performed as previously described (40, 44) with minor modifications. Primary antibodies included rabbit anti-LRRC17 (Novus catalog# NBP1-83309, RRID:AB_11001463, 1:400), mouse anti-TOMM20 (Abcam, catalog #ab56783, RRID:AB_945896), anti-glial fibrillary acidic protein (GFAP) (Millipore MAB3402 RRID:AB_94844, 1:500; and Dako, Z0334 RRID:AB_10013382, 1:500). Secondary antibodies were conjugated with either AlexaFluor-488 or AlexaFluor-568 (Thermo Fisher Scientific catalog #A-21206, also A21206 RRID:AB_2535792; Thermo Fisher Scientific catalog #A10037 RRID:AB_2534013). Nuclei were counterstained with DAPI. Images were taken using an A1RSi con-focal microscope system (Nikon) with 20×, 60× or 100× objectives.

### Isradipine treatment

*In vitro*, RTT MT astrocytes differentiated for 7 days were treated with 1 μM isradipine and incubated for 4 h (the same dose and duration as used in the primary HTS) before measurements. Neurons differentiated for 7 days were treated with 0.08 μM isradipine for 4 h and then measured by the JC-10 assay. This dose and treatment duration were selected after preliminary experiments testing multiple doses and treatment time points (Supplementary Figures S1, S2). All *in vitro* experiments included 6 independent biological replicates (separate differentiations), with 2–3 technical replicates performed for each biological sample, totaling 6 replicates for subsequent statistical analysis. *In vivo*, all mice are kept in the C57BL/6 background. Mice were housed in a facility with 12-h light/12-h dark cycle. Male *Mecp2^−/y^* mice (41) of 4–6 weeks of age were randomly assigned into two groups: one group receiving vehicle, the other receiving isradipine (Selleckchem catalog# S1662) and blinding was applied for behavioral scoring and data collection. The *in vivo* dosage was adopted based on two published studies (45, 46) validating this dose for isradipine in mouse neurological disease models, with reference to reported isradipine pharmacokinetics (including central nervous system tissue penetration). For each mouse assigned to isradipine group, isradipine was administered once daily via intraperitoneal injection at a dose of 3 mg/kg for as long as the animal was alive. Body weight and the semi-quantitative symptom score were recorded each week. To minimize bias, we standardized morphological staining protocols, performed blind analysis and used ImageJ for automated quantification.

### Statistical analysis

All data are presented as mean ± standard error of the mean (SEM) as appropriate for the statistical test used. The Shapiro–Wilk test was used to assess normality of distribution, and Levene’s test for homogeneity of variance. All data passed normality and homogeneity of variance tests. Parametric tests were used accordingly, including unpaired two-tailed Student’s *t*-test for comparisons between two groups, one-way ANOVA followed by appropriate *post-hoc* test for multiple-group comparisons, and two-way ANOVA followed by appropriate *post-hoc* analysis for factorial designs. *p* < 0.05 was considered statistically significant. Data were analyzed with Sigma Plot 13.0 and GraphPad Prism 10.0. *N* values are indicated in each figure legend and represent the number of independent biological replicates, individual cells, or experimental batches, as applicable to each assay.

## Results

### Screening of a library containing 1,134 FDA-approved drugs and an siRNA library against 336 genes to identify hits that rescue the MMP deficit in RTT astrocytes

**Figure 1:**
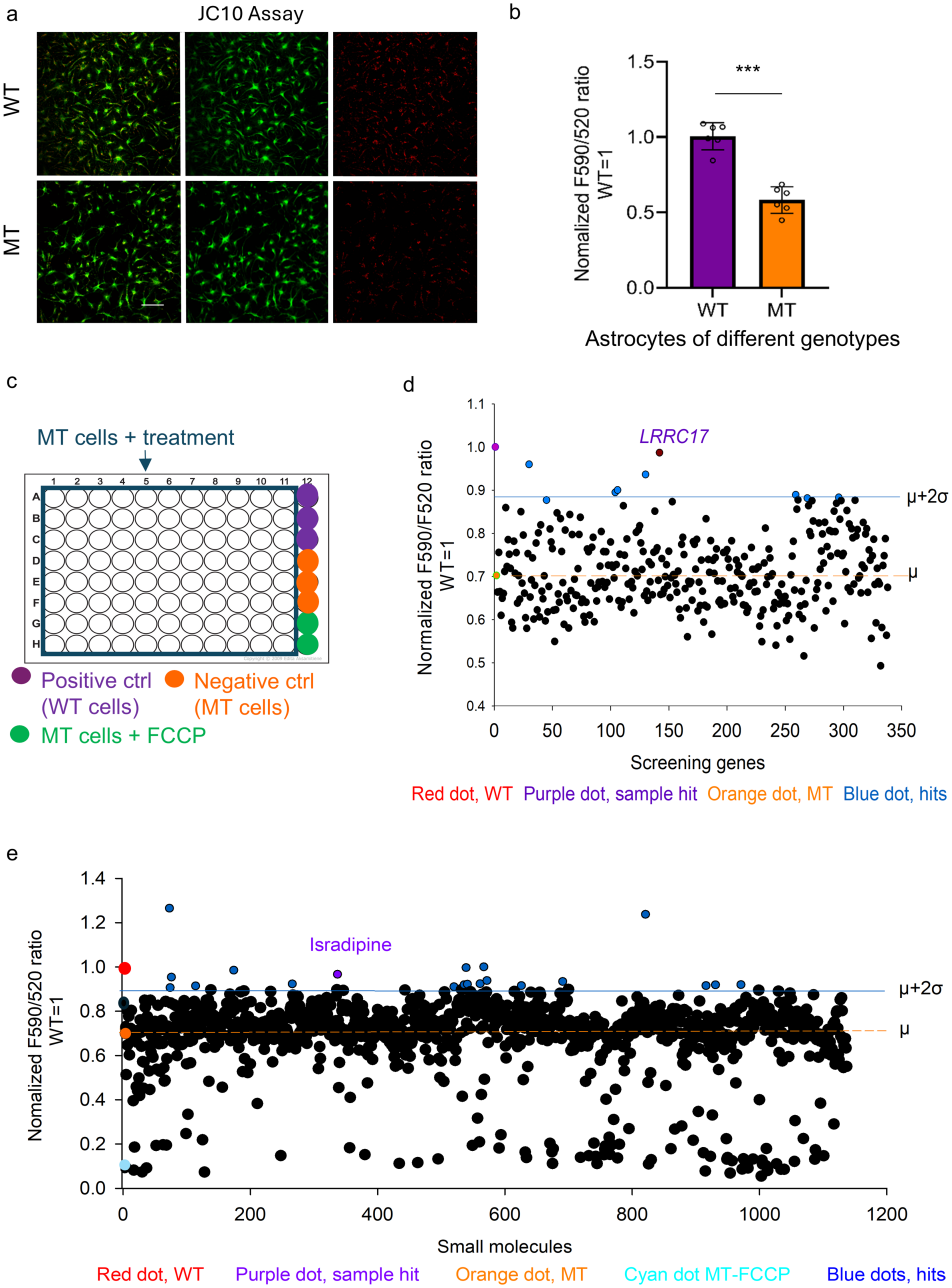
High-throughput screening identifying candidate drugs and candidate genes that improve mitochondrial health in RTT astrocytes. **(a)** Representative images of JC-10 fluorescence, *n* = 6 (*n* values refer to the number of independent differentiation batches), with 3 technical replicates per biological replicate, 5 images per slide. Left: overlay of green and red channels; middle; green channel; right: red channel in R294X WT and MT astrocytes. Scale bar = 100 μm. **(b)** Bar graph of normalized F590/520 ratio in R294X WT and MT astrocytes (^*^p < 0.05, ^**^p < 0.01, and ^***^p < 0.001, two-tailed *t*-test). **(c)** Plate layout of a representative 96-well plate used in the high-throughput screening. Purple wells: Positive control (WT cells). Orange wells: Negative control (MT cells). Green wells: MT cells + FCCP. Uncolored wells: MT cells + test treatment (either drugs or siRNAs). **(d)** Scatter plot showing the results (as quantified in F590/520 value) of the siRNA screening in R294X WT and MT astrocytes. 9 hit genes (Table 2), with F590/F520 at least 2 SDs above the mean, are shown in blue. **(e)** Scatter plot showing the results (as quantified in F590/520 value) of the small molecule screening. 20 hit compounds (Table 1), with F590/F520 2 SDs above the mean, are shown in blue.

To characterize the mitochondrial phenotypes in RTT astrocytes, we differentiated congenic WT and MT RTT iPSC lines into astrocytes as reported in our previous study (31, 41). This differentiation protocol is robust and consistent in that over 95% of the differentiated cells are positive for glial fibrillary acidic protein (GFAP). There was no difference in differentiation efficiency between the WT and MT iPSCs, astroglial progenitors and terminally differentiated astrocytes at each milestone steps of the differentiation procedure. The X chromosome inactivation status and allelic expression of *MECP2* in the astroglial progenitors and the terminally differentiated astrocytes were closely monitored to ensure correct genotype identification. Using the JC-10 assay, we measured the MMP in R294X WT and MT (Arginine 294 to stop codon, nonsense mutation, present in 5–6% of RTT patients) astrocytes and found that the R294X MT astrocytes showed a lower ratio of F590/F520 (Figures 1a,b).

While we are actively studying the cellular mechanisms underlying the mitochondrial phenotypes in RTT astrocytes, we recognized that the MMP phenotype can be easily assessed in live cells with the JC-10 assay and therefore is suitable for high-throughput screening. We first screened the Selleckchem FDA-approved Drug Library for compounds that can rescue the MMP deficit in R294X MT astrocytes. The library is chosen because all compounds contained in the library are drugs approved by the FDA. Briefly, R294X astrocytes were differentiated, and plated at a density of 1.5 × 10^4^ cells per well on 96-well plate pre-coated with Matrigel (BD Biosciences). The medium (DMEM/F12 containing 1% N2, 1× NEAA, 1× pen/strep, 10 ng/mL BMP4, 10 ng/mL LIF, and 10 ng/mL CNTF) was changed every 3 days. 7 days after plating, compounds from the Selleckchem FDA-approved Drug Library were added to each well at 1 μM and tested on duplicate 96-well plates. The 1 μM concentration for drug treatment was determined by using doxorubicin as a standard cytotoxicity control before the screen (i.e., compounds are not toxic to our cells at this concentration). Congenic WT and MT R294X astrocytes not exposed to any compound were used as positive and negative controls, respectively, on each screening plate (Figure 1c). Astrocytes treated with FCCP were used as the no-MMP control on each screening plate. 4 h after addition of compounds, cells were loaded with the JC-10 dye following manufacturer’s instructions, stained with Hoechst 33342 (to label the nuclei), and imaged using the Operetta High Content imaging platform (Perkin Elmer) at 20× magnification. Image analysis was performed using either the Harmony software (Perkin Elmer) or the Columbus software (Perkin Elmer). The F590/F520 ratio was generated as the quantitative measure for each well and averaged between the duplicate plates. To further ensure data quality and minimize plate-to-plate variation, intra-plate normalization was performed using WT controls for each plate. Screening in duplicate was determined by the Z′-factors. The F590/520 value of 2 standard deviations (SD) above the mean (*μ* + 2σ) was used as the cutoff to identify potential hits in the drug screening. Out of the 1,134 compounds in the library, we identified 20 positive hits (Figure 1e and Table 1). To prioritize candidates suitable for *in vivo* translation, we excluded compounds unable to cross the to cross the blood brain barrier (BBB) and validated the remaining 13 hits in multiple independent RTT neuronal lines. Hits that consistently rescued the primary mitochondrial phenotype were further characterized for additional mitochondrial functions, including ATP production, mitochondrial morphology, and spontaneous cytosolic calcium activity. Using this stepwise selection strategy (Supplementary Table S2), we eventually selected isradipine for *in vivo* validation in the *Mecp2^−/y^* mice because it rescued deficits in all the *in vitro* assays we performed.

**Table 1:** List of candidate drugs identified as hits in our drug screening.

Item name	Target	Brief description
Agomelatine	5-HT receptor	Serotonin(5-HT) (2C) receptors antagonist
Amfenac sodium	—	Analgesic anti-inflammatory drug
Arbidol HCl	Receptor	An antiviral treatment for influenza infection
Aripiprazole (Abilify)	5-HT receptor	Agonistic activity at dopamine D2 and 5-HT1A receptors
Blonanserin (Lonasen)	5-HT receptor	Dopamine D2 and serotonin 5-HT2 receptors antagonist
Cleviprex (Clevidipine)	Calcium channel	L-type calcium channel blocker
Deferiprone	—	An iron chelator
Domperidone (Motilium)	Dopamine receptor	Specific blocker of dopamine receptor
Duloxetine HC (Cymbalta)	5-HT receptor	Serotonin (5-HT) and norepinephrine (NE) reuptake inhibitors
Edaravone (MCl-186)	—	A potent antioxidant
Entacapone	COMT	Catechol-O-methyltransferase (COMT) inhibitor
Fluocinolone acetonide (Flucort-N)	Glucocorticoid receptor	Glucocorticoid receptor agonist
Isradipine (Dynacirc)	Calcium channel	Dihydropyridine calcium channel blockers
Ivabradine HC (Procoralan)	Funny channel	Selectively inhibit if current
Lomerizine HCl	Calcium channel	L-type and T-type calcium channel blocker
Megestrol acetate	Progesterone receptor	Progesterone receptor and glucocorticoid receptor agonist
Naftopidil (Flivas)	Adrenergic	Adrenergic receptor antagonist
Telbivudine (Sebivo, Tyzeka)	Nucleoside reverse transcriptase	Nucleoside analog, nucleoside reverse transcriptase inhibitor
Vinblastine	Microtubule	Inhibits microtubule formation
Vincristine	Microtubule	Inhibits assembly of microtubule structures

Using the same platform, a siRNA library containing siRNA against 336 genes upregulated in R294X MT astrocytes was also screened. 9 hits (Table 2) were identified to increase the F590/F520 ratio by at least 2 SDs above the mean (Figure 1d). Among these 9 candidate genes, *LRRC17* is the only gene encoding secreted protein. All these primary hits were subjected to independent secondary validation assays, and all candidates reproducibly improved MMP levels in R294X MT astrocytes (Supplementary Figures S3). Interestingly, one of the candidate genes identified in the screen is *TRPC4*, which was previously shown by our lab to play a significant role in abnormal calcium homeostasis in RTT astrocytes (41). In several cases (*LRRC17*, *TRPC4*, *KCNA2*) where high quality antibodies are available, we performed immunostaining and Western blotting experiments to validate the effectiveness of siRNA knockdown. In addition, these positive hits also exhibited significant rescue effects on mitochondrial functional deficits, including increased ATP production and restored normal mitochondrial morphological features (Supplementary Table S4). One gene of particular interest (*LRRC17*), encoding a secreted protein with strong effects on neuronal health, was selected for further mechanistic studies.

**Table 2:** List of candidate genes identified in our siRNA library screening.

Symbol	Gene name	Brief description
*ADGRL3*	Adhesion G protein-coupled receptor L3	Regulating astrocyte mitochondrial function and adhesion
*GABRQ*	Gamma-aminobutyric acid type A receptor subunit theta	Mediating astrocyte mitochondrial metabolic signaling
*HECTD2*	HECT domain E3 ubiquitin protein ligase 2	Modulating astrocyte mitochondrial homeostasis via ubiquitination
*KCNA2*	Potassium voltage-gated channel subfamily A member 2	Regulating astrocyte mitochondrial membrane potential
*KIF27*	Kinesin family member 27	Modulating astrocyte mitochondrial trafficking and distribution
*LRRC17*	Leucine-rich repeat-containing 17	Linked to astrocyte mitochondrial structure and function
*PABPC5*	Poly(A) binding protein cytoplasmic 5	Regulating astrocyte mitochondrial RNA metabolism
*PCDHB5*	Protocadherin beta 5	Mediating astrocyte adhesion and mitochondrial metabolic coupling
*TRPC4*	Transient receptor potential cation channel subfamily C member 4	Regulating astrocyte mitochondrial Ca^2+^ homeostasis and energy metabolism

### Isradipine rescues mitochondrial deficits in RTT astrocytes and neurons *in vitro* and provides preliminary evidence of improved characteristic RTT phenotypes in *Mecp2* knockout mice *in vivo*

**Figure 2:**
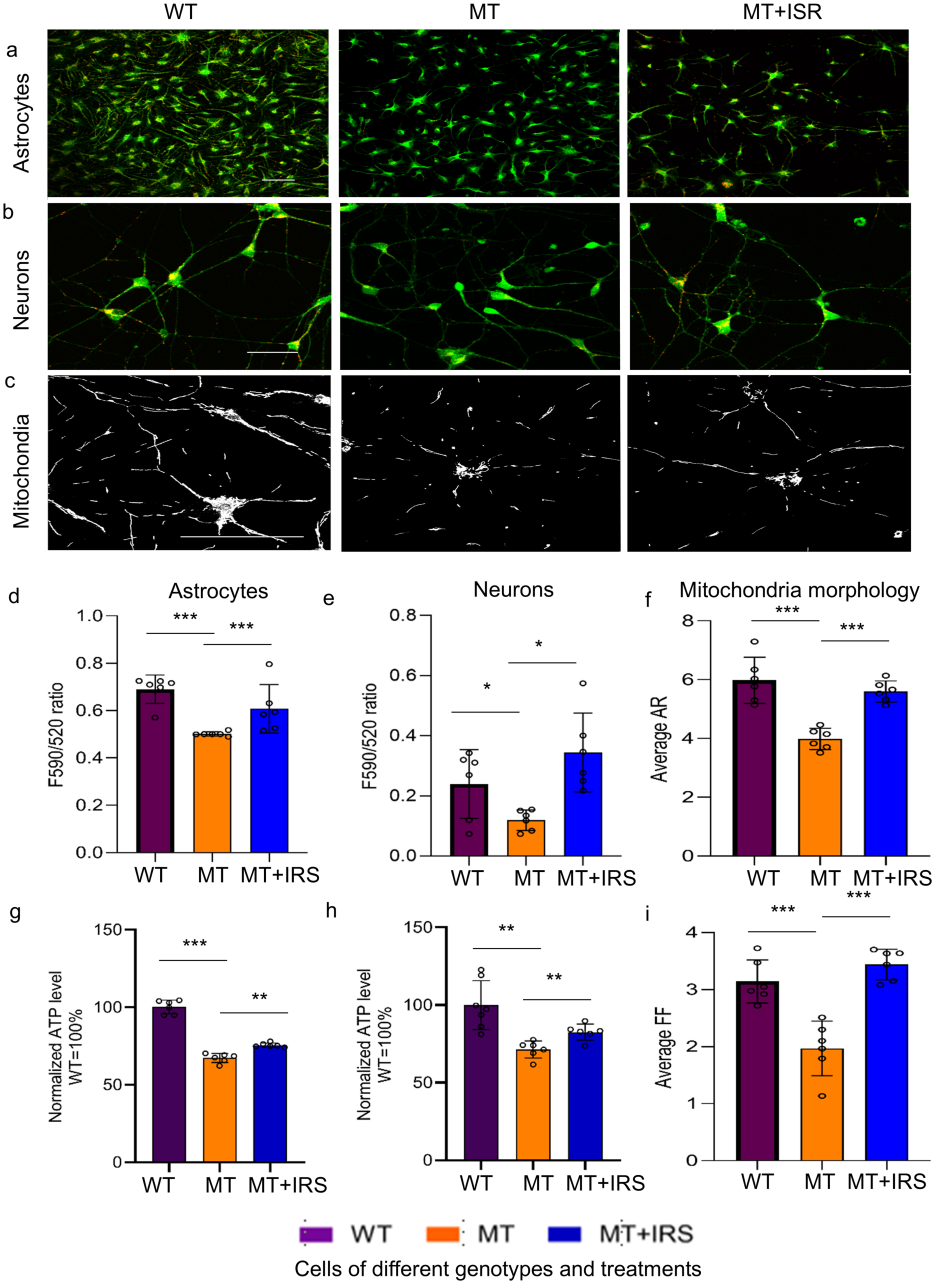
Isradipine treatment improves mitochondrial function in RTT astrocytes and RTT neurons *in vitro*. **(a)** Representative images of JC-10 fluorescence (overlay of green and red channels) in H9 WT astrocytes, T158M astrocytes, and T158M MT astrocytes treated with 1 μM isradipine (MT + ISR). Scale bar = 50 μm. **(b)** Representative images of JC-10 fluorescence (overlay of green and red channels) in H9 WT neurons, T158M neurons, and T158M neurons treated with 1 μM isradipine (MT + ISR). Scale bar = 20 μm. **(c)** Representative images of Rh123 staining in V247fs WT astrocytes, V247fs MT astrocytes, and V247fs MT astrocytes treated with 1 μM isradipine (MT + ISR). Scale bar = 10 μm. **(d)** Quantification of the F590/520 ratio in H9 WT, V247fs WT astrocytes, KO, T158M, V247fs MT astrocytes, and MT(KO) astrocytes treated with 1 μM isradipine (MT + ISR). **(e)** Quantification of the F590/520 ratio in T158M WT neurons, T158M neurons, and T158M neurons treated with 1 μM isradipine (MT + ISR). **(f)** Quantification of the aspect ratio (AR) based on rhodamine123 staining in V247fs WT astrocytes, V247fs MT astrocytes, and V247fs MT astrocytes treated with 1 μM isradipine (MT + ISR). **(g)** Quantification of ATP production in V247fs WT astrocytes, V247fs MT astrocytes, and V247fs MT astrocytes treated with 1 μM isradipine (MT + ISR). **(h)** Quantification of ATP production in V247fs WT neurons, V247fs MT neurons, and V247fs MT neurons treated with 1 μM isradipine (MT + ISR). **(i)** Quantification of the form factor (FF) based on Rh123 staining in V247fs WT astrocytes, V247fs MT astrocytes, and V247fs MT astrocytes treated with 1 μM isradipine (MT + ISR). All experiments for isradipine treatment included 6 independent biological replicates (separate cell differentiations) with 2–3 technical replicates per biological sample, 3–6 images per slide. Data was analyzed by one-way ANOVA with Tukey’s *post-hoc* test. All bar graphs in this figure are expressed as the mean ± s.e.m. ^*^p < 0.05, ^**^p < 0.01, ^***^p < 0.001.

**Figure 3:**
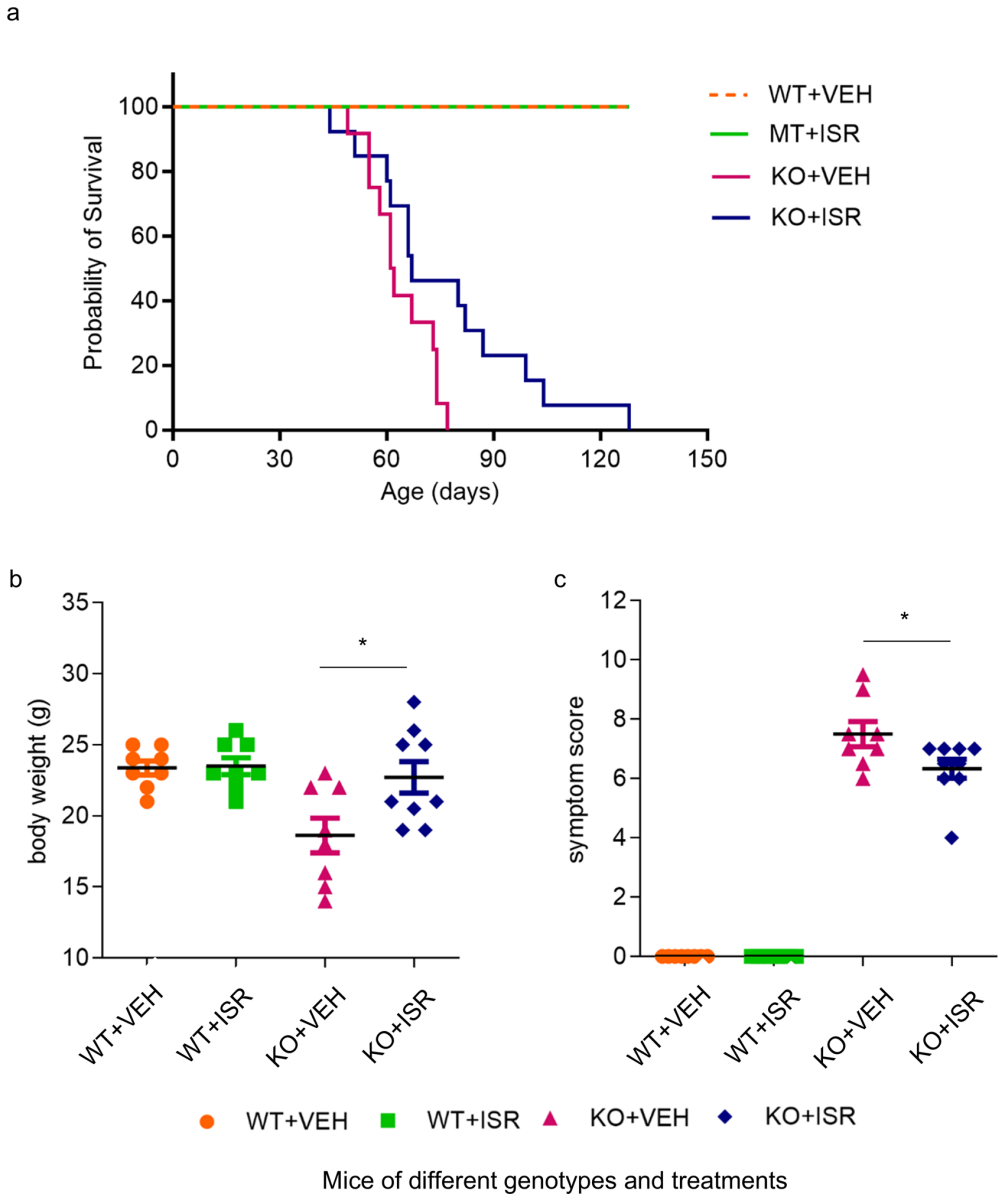
Isradipine improves characteristic phenotypes and overall health in *Mecp2* knockout mice. **(a)** Survival curves of *Mecp2^KO^* mice receiving vehicle (MT + VEH, *n* = 8), *Mecp2^KO^* receiving 3 mg/kg isradipine daily (MT + ISR, *n* = 9), WT mice receiving vehicle (WT + VEH, *n* = 8), and WT mice receiving 3 mg/kg isradipine daily (WT + ISR, *n* = 8). Survival probability was analyzed by the log-rank test, while body weight and symptom score were analyzed by one-way ANOVA with Tukey’s *post-hoc* test. **(b)** Body weight of *Mecp2^KO^* receiving vehicle (MT + VEH, *n* = 8), *Mecp2^KO^* mice receiving 3 mg/kg isradipine daily (MT + ISR, *n* = 9), WT mice receiving vehicle (WT + VEH, *n* = 8), and WT mice receiving 3 mg/kg isradipine daily (WT + ISR, *n* = 8). Data are expressed as the mean ± s.e.m. ^*^p < 0.05, ^**^p < 0.01, ^***^p < 0.001. **(c)** Semi-quantitative symptom scores of *Mecp2^KO^* mice receiving vehicle (MT + VEH, *n* = 8), *Mecp2^KO^* mice receiving 3 mg/kg isradipine daily (MT + ISR, *n* = 9), WT mice receiving vehicle (WT + VEH, *n* = 8), and WT mice receiving 3 mg/kg isradipine daily (WT + ISR, *n* = 8). Data are expressed as the mean ± s.e.m. ^*^p < 0.05, ^**^p < 0.01, ^***^p < 0.001.

While all the candidate drug hits identified from the screen passed independent validation, we selected isradipine, an L-type calcium channel blocker, for further investigation. This choice was in part motivated by our previously observation that calcium homeostasis is dysregulated in RTT astrocytes (41), making calcium modulators particularly relevant targets. In a series of *in vitro* validation experiments, isradipine demonstrated efficacy in correcting several mitochondrial deficits in both mutant astrocytes and neurons (Figure 2). As measured by JC-10 staining, isradipine normalized MMP in both T158M mutant astrocytes (Figures 2a,d) and neurons (Figures 2b,e). We also repeated the MMP rescue experiment in astrocytes of additional RTT genotypes (*MECP2^KO^*, *MECP2^V247fs^* mutant) in multiple independent differentiations, and observed similar rescuing effects. For quantitation in Figure 2d, all mutant lines (KO, T158M, V247fs) were grouped as MT, and all corresponding wild-type lines (H9 for KO/T158M, matched WT for V247fs) were pooled as WT. In addition to consistently rescuing the MMP deficit, isradipine normalized the morphology of mitochondria in MT astrocytes (Figure 2c) as quantified by aspect ratio (AR) (Figure 2f) and form factor (FF) (Figure 2i). Finally, isradipine significantly improved ATP production in both MT astrocytes (Figure 2g) and MT neurons (Figure 2h). *In vivo*, isradipine provided preliminary evidence of improved survival in *Mecp2^−/y^* mice compared to the vehicle-treated group, with a *p*-value of 0.016 (Figure 3a, log rank test; median survival: 68 days in the isradipine-treated group vs. 60 days in the vehicle-treated group), normalized body weight in *Mecp2* knockout mice (Figure 3b), and provided preliminary evidence of improved semi-quantitative symptom score (covering characteristic RTT phenotypes of mobility, gait, hindlimb clasping, tremor, breathing, and general condition) of *Mecp2* knockout mice (Figure 3c).

### siRNA knockdown of *LRRC17* rescues abnormal mitochondrial morphology, ATP production deficit, and abnormal calcium activity in RTT astrocytes

**Figure 4:**
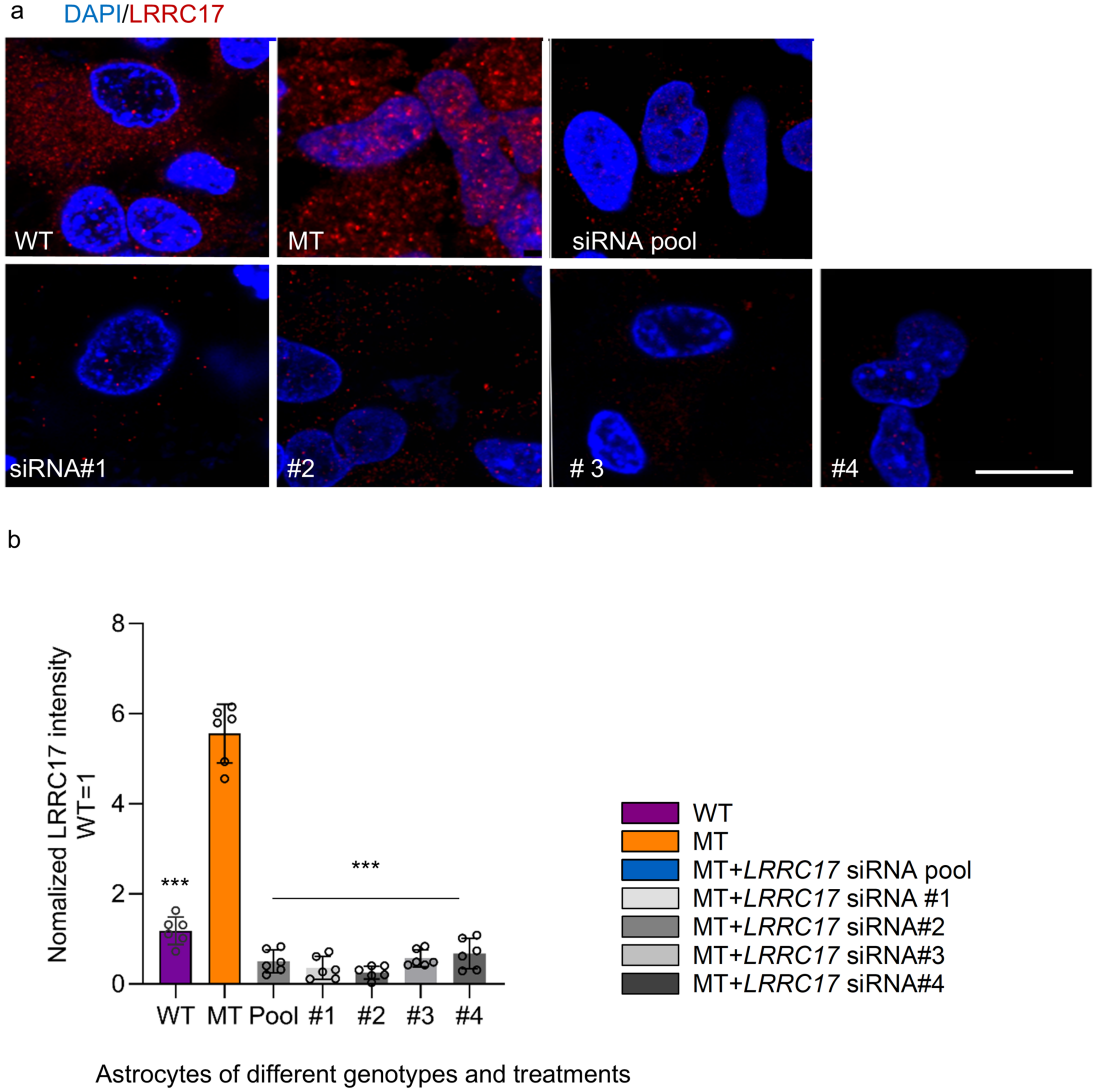
Quantification of the level of LRRC17 protein by immunostaining in astrocytes of different genotype/treatment groups. **(a)** Representative confocal microscopy images of LRRC17 immunoreactivity (red) and DAPI staining (blue) in R294X WT astrocytes, MT astrocytes, and MT astrocytes transfected with siRNA pool and each of the 4 single siRNA against LRRC17. Scale bar = 10 μm. **(b)** Bar graph showing quantification of LRRC17 immunoreactivity in R294X WT astrocytes, MT astrocytes, and MT astrocytes transfected with siRNA pool and each of the 4 single siRNA against LRRC17. At least 12 total replicates analyzed from 6 separate differentiations of each cell line (*n* = 6), 3–6 images per slide. Normalized LRRC17 intensity was analyzed by one-way ANOVA with Tukey’s *post-hoc* test to compare differences among WT, MT, and MT + LRRC17 siRNA groups. (^*^p < 0.05 and ^***^p < 0.001).

**Figure 5:**
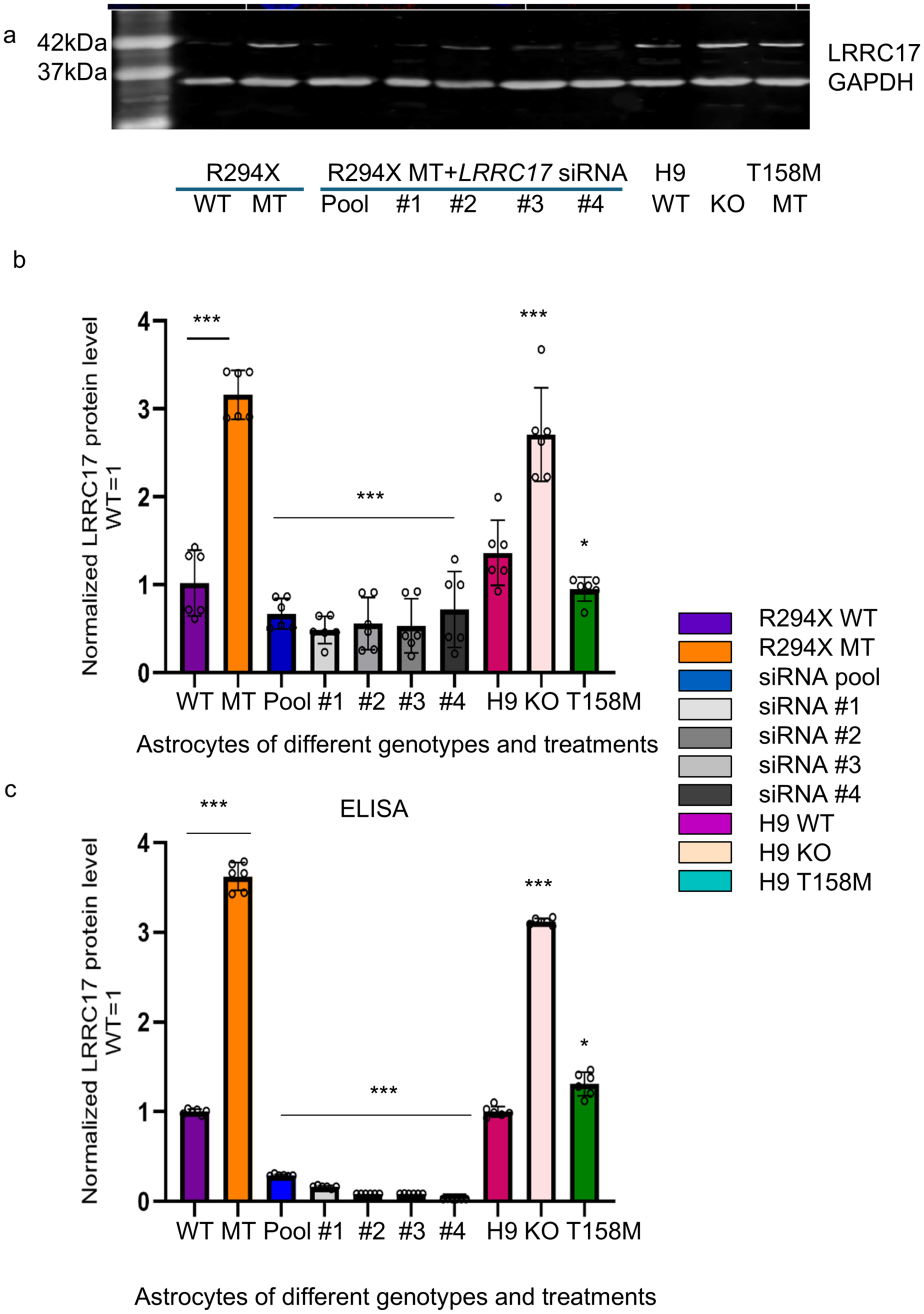
Quantification of LRRC17 level in whole cell lysate by Western blot and quantification of LRRC17 level in the astrocyte-conditioned medium by ELISA in astrocytes of different genotype/treatment groups. **(a)** Representative Western blot images of the detection of LRRC17 protein in whole cell lysates from R294X astrocytes and the congenic R294X WT control astrocytes, R924X MT astrocytes transfected with siRNA pool and each of the 4 single siRNA against *LRRC17*, astrocytes differentiated from parental H9 hESCs (H9-WT), astrocytes differentiated from H9 ESCs without MECP2(*MECP2^KO^*), astrocytes differentiated from H9 ESCs expressing MECP2 with the T158M mutation and H9 WT astrocytes. **(b)** Bar graph showing quantification of Western blot analysis. 12–18 total replicates from 6 separate differentiations from each cell line were included in the analysis (*n* = 6). **(c)** Bar graph showing ELISA quantification of secreted LRRC17 protein level in the astrocyte conditioned medium. 18 total replicates from 6 separate differentiations of each cell line were included in the analysis (*n* = 6). Normalized LRRC17 protein levels (Western blot and ELISA) were analyzed by one-way ANOVA with Tukey’s *post-hoc* test to compare differences among R294X WT, R294X MT, R294X MT + *LRRC17* siRNA, H9 WT, KO and T158M, groups. (^*^p < 0.05 and ^***^p < 0.001).

**Figure 6:**
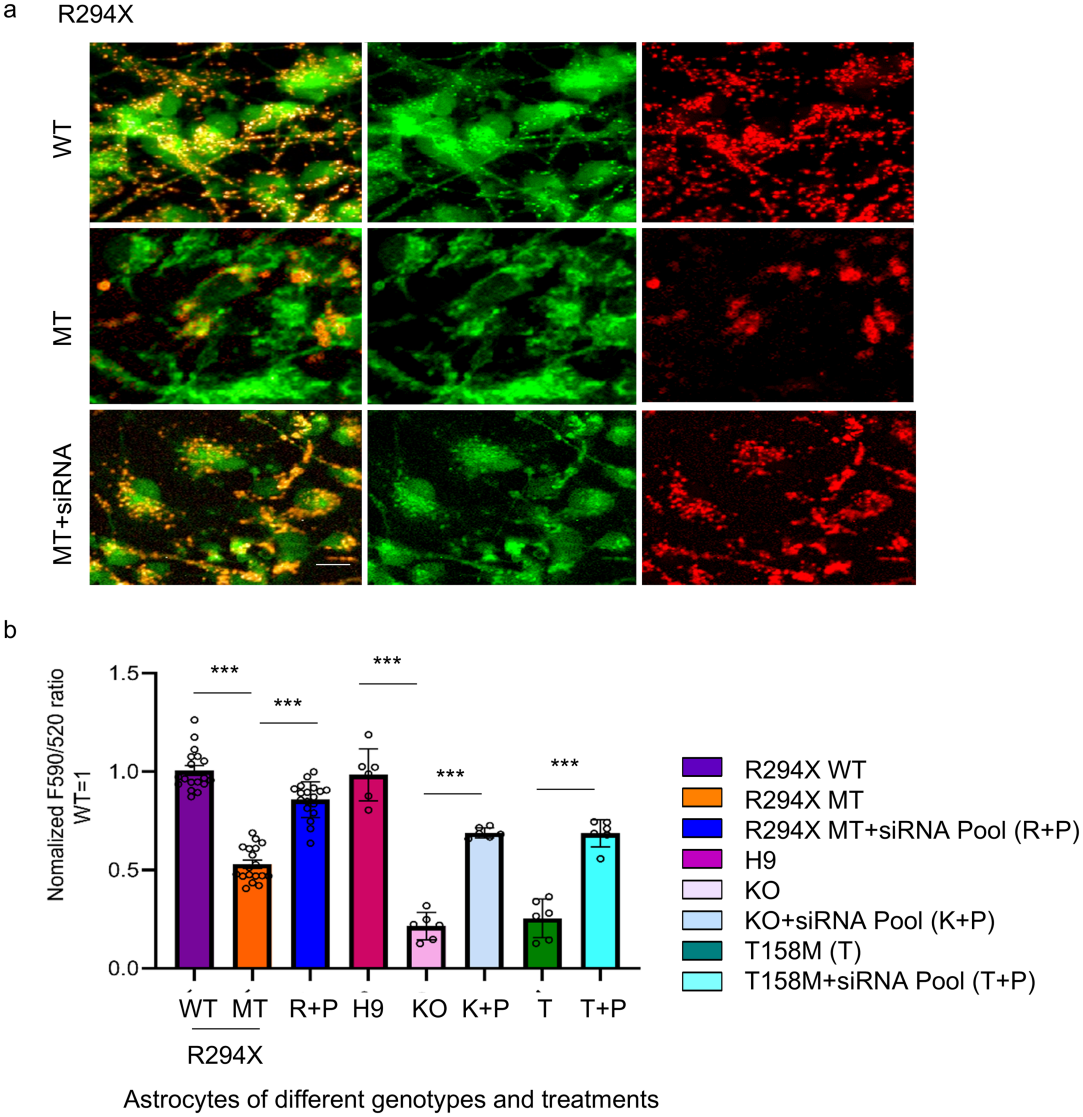
Validation of the rescue of the MMP deficit in RTT astrocytes by siRNA-mediated knockdown of *LRRC17*. **(a)** Representative confocal microscopy images of JC-10 fluorescence (left: overlay of green and red channels; middle: green channel; right: red channel) in R294X WT astrocytes, MT astrocytes, and MT astrocytes transfected with a pool of siRNA against *LRRC17* (MT + siRNA). Scale bar = 10 μm. **(b)** Quantification of the F590/520 ratio in R294X, T158M and KO astrocytes, and astrocytes transfected with a pool of siRNA against *LRRC17* (MT/KO + siRNA), corresponding WT cell lines ware set as the control. (^***^p < 0.001).

**Figure 7:**
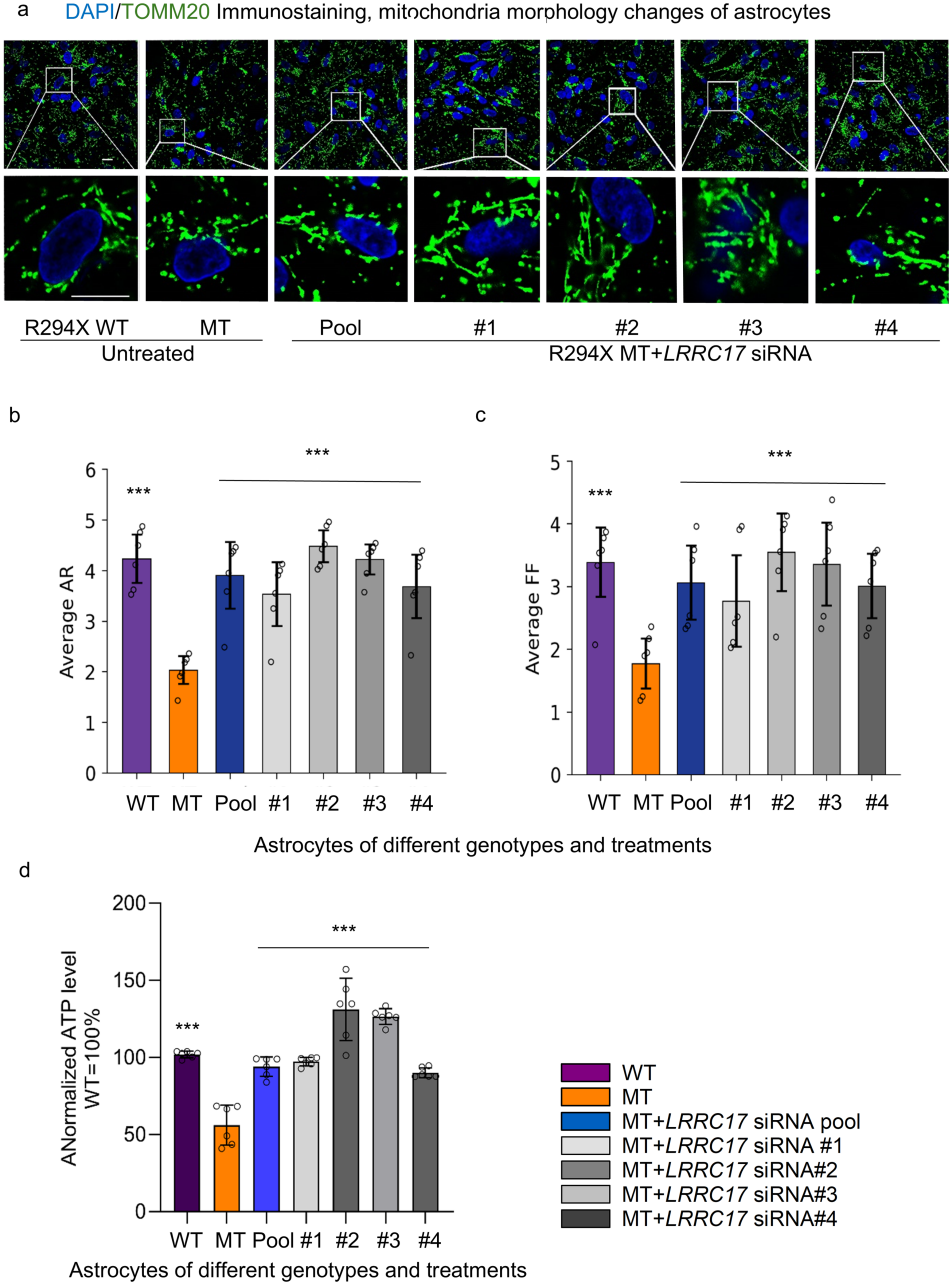
Treatment with *LRRC17* siRNA rescues additional mitochondrial phenotypes in RTT astrocytes. **(a)** Representative confocal microscopy images of mitochondrial morphology labeled by TOMM20 immunoreactivity (green) immunoreactivity (red) and DAPI staining (blue) in R294X WT astrocytes, MT astrocytes, and MT astrocytes transfected with siRNA pool and each of the 4 single siRNA against *LRRC17* (top: 20× magnification, bottom: 100× magnification view a selective region of interest in the 20× image). Scale bars = 10 μm. **(b,c)** Bar graph showing mitochondrial morphology analysis as quantified by the value of aspect ratio (AR, **b**) and form factor (FF, **c**) in R294X wild WT astrocytes, MT astrocytes, and MT astrocytes transfected with siRNA pool and each of the 4 single siRNA against *LRRC17*. Comparisons between two groups were determined by the unpaired Student’s *t*-tests. **(d)** Quantification of ATP level was significantly increased after *LRRC17* siRNA treatment in *R294X* iPSCs derived astrocytes. R294X WT astrocytes, MT astrocytes, and MT astrocytes transfected with siRNA pool and each of the 4 single siRNA against *LRRC17*. Biological replicates: an independent differentiation batch of astrocytes. Data represents 6 independent biological replicates. For each biological replicate, 2 technical replicates (wells) were analyzed, with 2–3 fields of view captured per well. Average AR, average FF, and normalized ATP levels were analyzed by one-way ANOVA with Tukey’s *post-hoc* test to compare differences among R294X WT, R294X MT, and R294X MT + *LRRC17* siRNA groups. (^***^p < 0.001).

**Figure 8:**
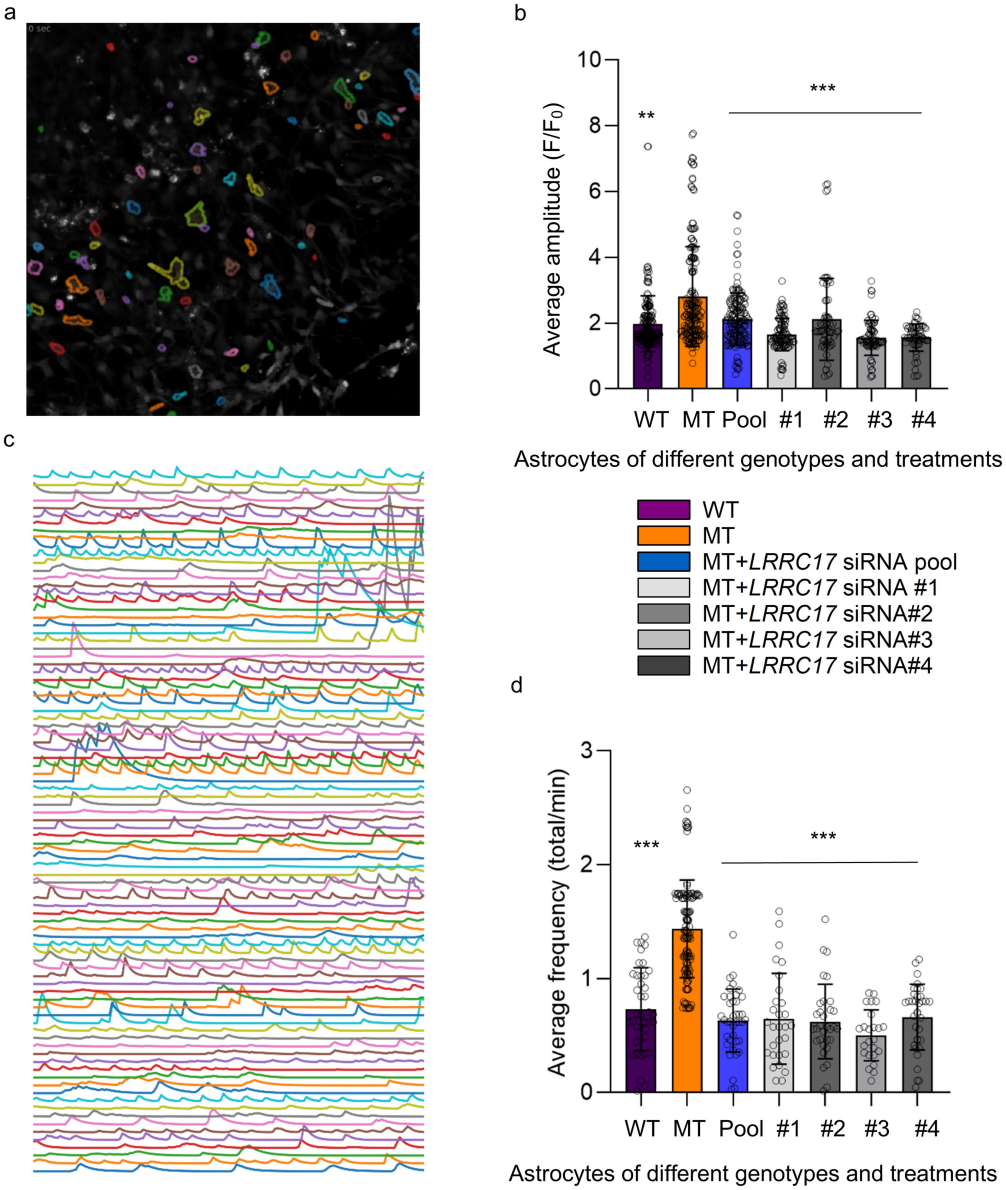
Treatment with *LRRC17* siRNA rescues the phenotype of abnormal spontaneous cytosolic Ca^2+^ activity in RTT astrocytes. **(a)** A representative confocal image of a frame of Fluo-4 fluorescence (as the proxy of spontaneous cytosolic Ca^2+^ activity) with many astrocytes circled as region of interest (ROI). **(b)** Quantification of the average amplitude of all identified spontaneous cytosolic Ca^2+^ activity in R294X WT astrocytes, MT astrocytes, and MT astrocytes transfected with siRNA pool and each of the 4 single siRNA against *LRRC17*. **(c)** Traces of Fluo-4 fluorescence over time from each of the ROIs in the image in **a**. **(d)** Quantification of the average frequency of all identified spontaneous cytosolic Ca^2+^ activity in R294X WT astrocytes, MT astrocytes, and MT astrocytes transfected with siRNA pool and each of the 4 single siRNA against *LRRC17*. (^**^p < 0.01, ^***^p < 0.001).

Among the identified candidate genes, we selected *LRRC17*, the only one encoding a secreted protein, for further studies, because previously work in our lab and others reported RTT astrocyte-conditioned medium has negative impact on neurons (31). To validate the elevated LRRC17 level in MT astrocytes, and reduction of LRRC17 level in MT astrocytes by both the original pool of siRNA and each individual siRNA against *LRRC17*, immunostaining was used to quantify LRRC17 immunoreactivity in cultured WT astrocytes, R294X astrocytes, and R294X astrocytes treated with siRNA against *LRRC17* (Figure 4). In addition, Western blot of whole cell lysates was used to quantify LRRC17 protein level of cultured astrocytes of each genotype and treatment group (Figures 5a,b). Since LRRC17 is a secreted protein, ELISA assay was used to examine LRRC17 in astrocyte-conditioned medium from astrocytes of different genotype and treatment groups (Figure 5c). The consistent results across immunostaining, Western blot, and ELISA not only confirmed the upregulation of LRRC17 in RTT astrocytes but also validated the specificity of the siRNA against *LRRC17*. In addition to R294X astrocytes (Figure 6a), astrocytes expressing no *MECP2* (*MECP2^KO^*) or other RTT causing mutations (T158M) were included to demonstrate the overexpression of LRRC17 is a common dysregulation associated with *MECP2* loss-of-function mutations (Figure 6b). After confirming the rescue of the MMP phenotype in RTT astrocytes by siRNA against *LRRC17*, we extended our analysis to look at mitochondrial structure. To better visualize and quantify mitochondrial morphology, we labeled astrocytes with TOMM20 (Figure 7a), which localized specifically to the mitochondria. Detailed quantification of the shape of TOMM20 immunoreactivity revealed significantly reduced aspect ratio (AR) (Figure 7b) and form factor (FF) (Figure 7c) in R294X astrocytes compared with WT astrocytes. These phenotypes were rescued by siRNA against *LRRC17* (Figures 7b,c). In addition, ATP production, another important function of the mitochondria, was significantly decreased in R294X astrocytes compared with WT, which was rescued in by siRNA against *LRRC17* (Figure 7d). Finally, using the green-colored calcium sensitive dye Fluo-4, we examined spontaneous cytosolic calcium (Ca^2+^) dynamics in live MT and WT control astrocytes [Figures 8a,c, for representative videos, see (Supplementary videos 1–2)]. Consistent with our previous report (41), a significantly higher percentage of MT astrocytes (28 ± 4% in MT vs. 16 ± 3% in WT, *n* = 18 randomly selected fields in each genotype/treatment group, *p* < 0.01) showed spontaneous oscillation in cytosolic Ca^2+^ levels. In each astrocyte showing spontaneous oscillation, we further quantified the amplitude and frequency of Ca^2+^ transients. The MT cells had a significantly higher amplitude (Table 3 and Figure 8b) and significantly higher frequency (Figure 8d) than their WT controls. To determine whether the observed abnormal cytosolic Ca^2+^ activities were related to elevated LRRC17 in these RTT MT astrocytes, we transfected MT astrocytes with *LRRC17* siRNA pool or each of the four single siRNA. Three days after the transfection, more than 90% of the cells were siRNA positive in each transfection group. The *LRRC17* siRNA pool and four single siRNA treated T158M astrocytes had significantly lower amplitude (Figure 8b) and frequency (Figure 8d) of cytosolic Ca^2+^ activities than the untreated MT astrocytes, suggesting the upregulated expression of LRRC17 contributes to abnormal Ca^2+^ activity in RTT MT astrocytes (for representative videos, see [Supplementary videos 3–7]).

**Table 3:** Calcium activity data.

Groups	No. of cells	Amplitude (*F*/*F*_0_)	Frequency (total/min)	Amplitude *p*-values (vs. MT)	Frequency *p*-values (vs. MT)
WT	224	1.91 ± 0.07	0.73 ± 0.06	*p* ≤ 0.01	*p* ≤ 0.001
MT	363	2.95 ± 0.18	1.44 ± 0.04	—	—
MT + *LRRC17* siRNA pool	204	1.67 ± 0.06	0.63 ± 0.04	*p* ≤ 0.001	*p* ≤ 0.001
MT + *LRRC17* siRNA #1	309	1.71 ± 0.05	0.63 ± 0.07	*p* ≤ 0.001	*p* ≤ 0.001
MT + *LRRC17* siRNA #2	178	2.01 ± 0.15	0.60 ± 0.06	*p* ≤ 0.001	*p* ≤ 0.001
MT + *LRRC17* siRNA #3	241	1.63 ± 0.07	0.50 ± 0.05	*p* ≤ 0.001	*p* ≤ 0.001
MT + *LRRC17* siRNA #4	214	1.36 ± 0.11	0.66 ± 0.05	*p* ≤ 0.001	*p* ≤ 0.001

Ca^2+^ amplitude and frequency were quantified at the single-cell level. Each cell was treated as one independent observation. Importantly, cells were obtained from a minimum of three independent differentiations per group. Differentiations were performed independently on separate days.

### The morphology of wild type neurons is sensitive to the LRRC17 protein level in the culture medium

**Figure 9:**
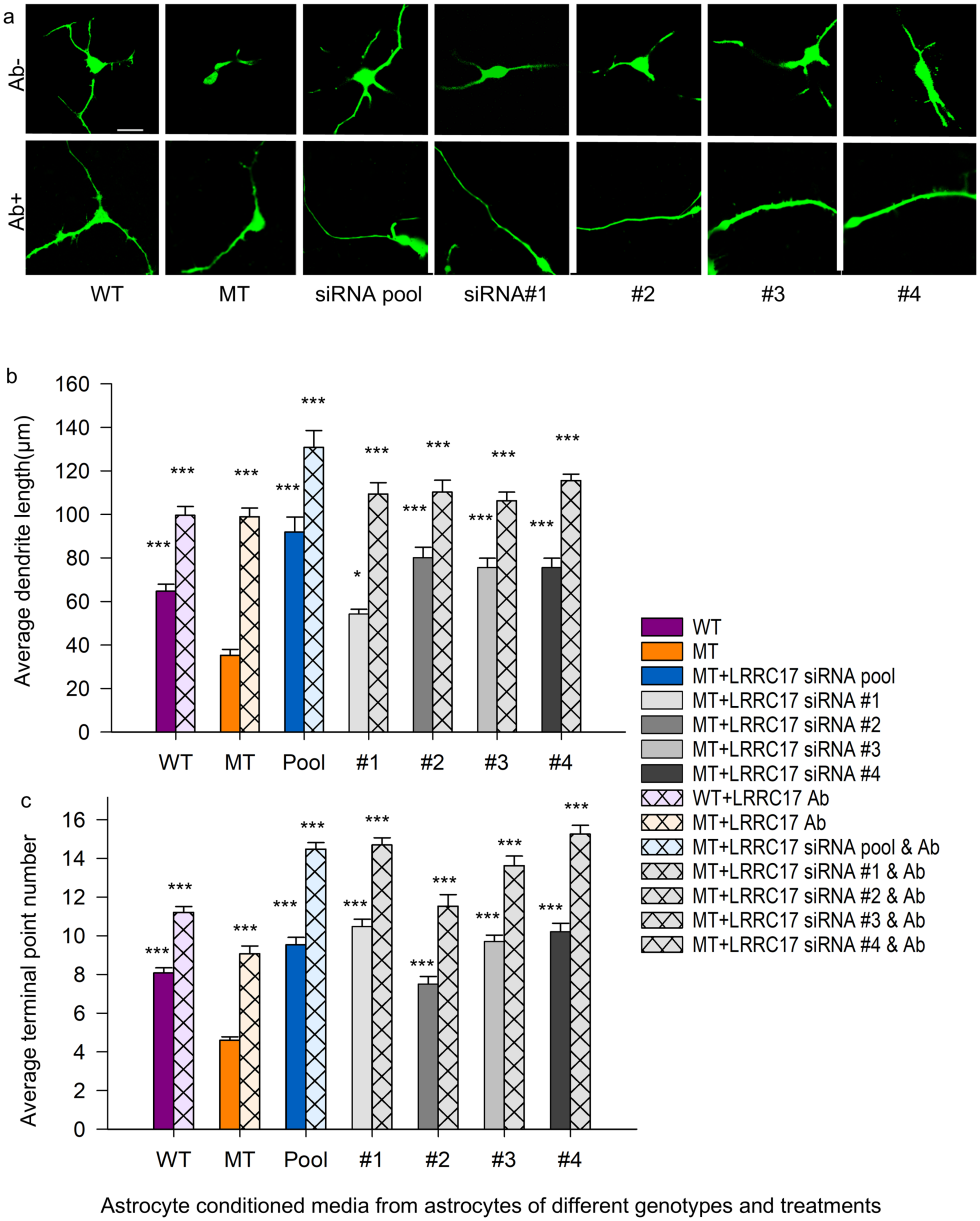
The non-cell autonomous negative impact of RTT astrocytes on neuronal morphology is dependent on the presence of LRRC17 in the astrocyte-conditioned medium. Neuronal morphology was evaluated following treatment with astrocyte *LRRC17*-siRNA-conditioned medium. **(a)** Representative images of H9-GFP wild-type neurons cultured in the medium conditioned by R294X WT astrocytes, MT astrocytes, and MT astrocytes transfected with siRNA pool and each of the 4 single siRNA against *LRRC17* (**a**: in the absence of LRRC17 neutralizing antibody, Ab^−^; **b**; in the presence of LRRC17 neutralizing antibody, Ab^+^). Scale bar = 10 μm. **(b,c)** Bar graph showing neuronal morphology analysis quantified in the average total dendrite length (top) and the average number of terminal points in GFP labeled wild type neurons cultured in the medium conditioned by R294X WT astrocytes, MT astrocytes, and MT astrocytes transfected with siRNA pool and each of the 4 single siRNA against *LRRC17*, either in the absence or presence of a LRRC17 neutralizing antibody. 182–196 neurons from each group were included in the analysis. Astrocyte-conditioned medium was collected from 6 separate differentiations of each astrocyte lines. Neurons were used from 6 separate differentiations from NP cells. For each biological replicate, at least 2 technical replicates (wells) were analyzed, with 3–6 fields of view captured per well. Comparisons were analyzed by two-way ANOVA followed by Holm-Sidak *post-hoc* test. In the figure, asterisks on the left of each pair indicate significance compared with the MT (Ab^−^) group, whereas asterisks on the right indicate significance between the Ab^+^ and Ab^−^ conditions within the same treatment group (^***^p < 0.001).

**Figure 10:**
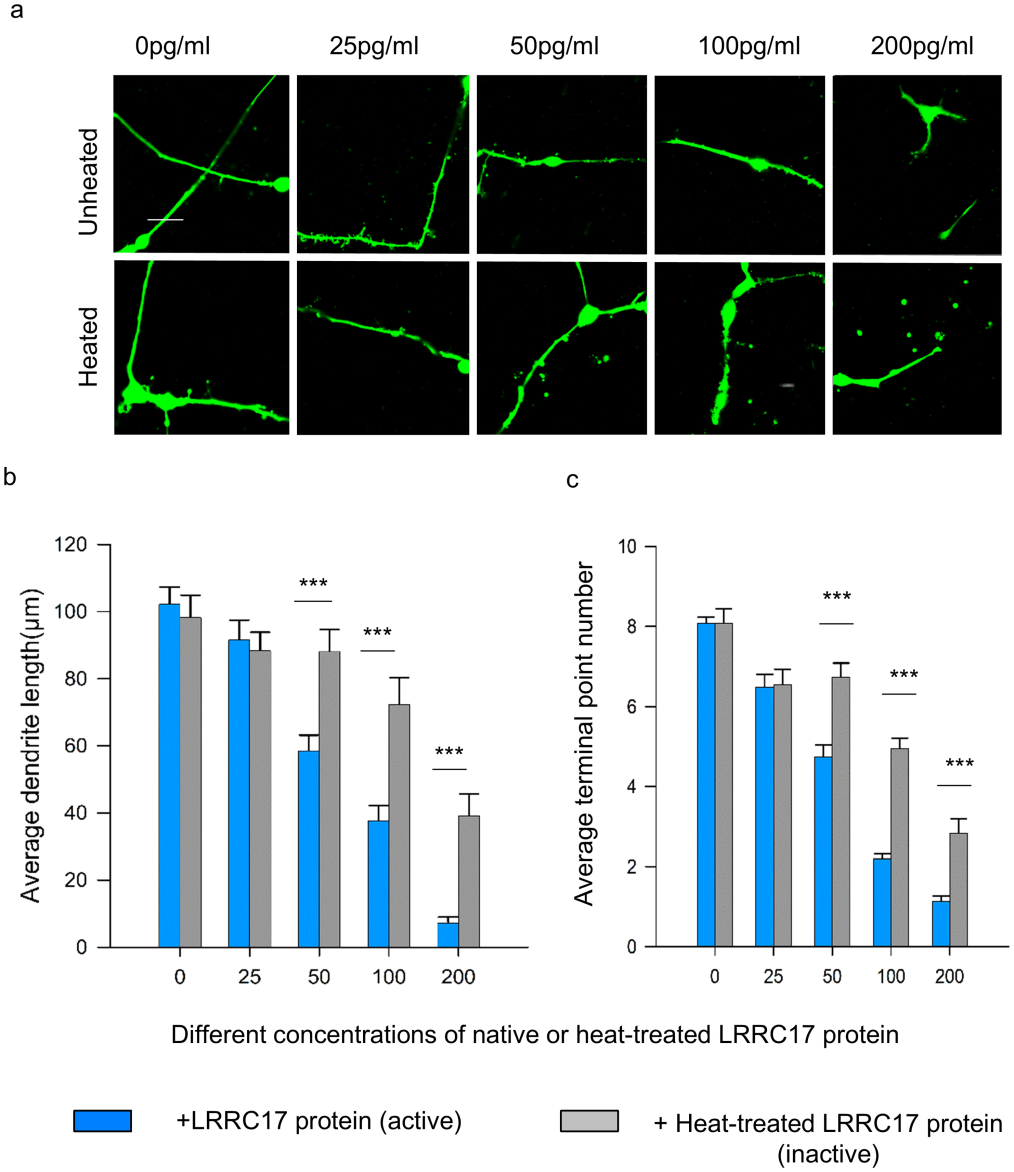
Exogenous recombinant full-length human LRRC17 protein in the culture medium shows dose-dependent negative impact on neuronal morphology. Neuronal morphology was assessed upon dose-dependent treatment with recombinant LRRC17 protein. **(a)** Representative confocal microscopy images H9-GFP neurons cultured in the medium containing different doses of native (top: untreated) or heat-inactivated (bottom: heated) the full-length (72.6 kDa) recombined human LRRC17 protein. Scale bar = 10 μm. **(b,c)** Bar graph showing neuronal morphology analysis quantified in the average total dendrite length (top) and the average number of terminal points in GFP labeled wild type neurons cultured in the medium containing different doses of native (active) or heat-inactivated (inactive) recombinant full-length human LRRC17 protein. 59–184 neurons of each treatment group differentiated from 6 differentiations were included in the analysis. For each biological replicate, at least 2 technical replicates (wells) were analyzed, with 3–6 fields of view captured per well. Statistical analysis was performed using two-way ANOVA to evaluate the effects of LRRC17 protein treatment and dose on dendritic morphology. Interaction between treatment and dose was also assessed. *Post-hoc* comparisons were performed using Holm-Sidak’s multiple comparisons test. (^***^p < 0.001).

Following confirmation of LRRC17 protein expression (IHC/Western blot) and mitochondrial functional rescue (JC-10, morphology, ATP, we next assessed its effect on neuronal morphology via astrocyte (with or without siRNA treatment)-conditioned medium. To investigate whether elevated level of LRRC17 in RTT MT astrocytes have any non-cell autonomous effect on wild type neurons, we differentiated human embryonic stem cells expressing the green fluorescence protein (H9-GFP hESCs) into forebrain neurons (H9-GFP neurons), and cultured the H9-GFP neurons in medium conditioned by WT astrocytes, untreated MT astrocytes, MT astrocytes treated with siRNA pool and individual siRNA against *LRRC17* (Figure 9a, top panel). Sholl analysis of these GFP-positive neurons revealed significantly reduced dendritic arborization and total neurite length in neurons cultured in MT astrocytes-conditioned when compared with neuron cultured in the WT astrocyte-conditioned medium (Figures 9b,c). These deficits were rescued when the MT astrocytes were treated with siRNA against *LRRC17* (Figure 9b). More importantly, neuronal morphology was significantly improved when a LRRC17 neutralizing antibody (Ab) was added in each of the astrocyte-conditioned medium (Figure 9a, bottom panel, and Figures 9b,c), suggesting LRRC17 is among astrocyte-secreted factors that mediate their non-cell autonomous impact on neuronal morphology. To examine whether ectopic LRRC17 is sufficient to confer negative impact on neuronal morphology, we added full-length recombinant human LRRC17 protein at different concentration directly in culture media and examined the morphology of H9-GFP neurons, using heat-inactivated LRRC17 protein as a negative control (Figure 10a). Results from Sholl analysis revealed a dose-dependent reduction of dendritic complexity and total neurite length in neurons exposed to LRRC17 protein at the concentration 50 pg/mL and higher (Figures 10b,c). Together, these results strongly implicate LRRC17 as a mediator of the non-cell autonomous effect of RTT astrocytes on neurons.

## Discussion

Since the identification of *MECP2* mutations as the cause of RTT, both mouse models (39, 45, 46) and RTT patient-specific induced pluripotent stem cell (iPSC) models (38, 47, 48) have been successfully generated. While studies employing these models have significantly advanced our understanding of the molecular and cellular mechanisms of the disease (49), they have not been used as high-throughput screening platforms to identify novel therapeutic targets. At the same time, although many studies suggest that compromised mitochondria health appears to be a common feature in multiple cell types in RTT patients and RTT models, the cellular and molecular mechanisms underlying mitochondrial dysfunction in RTT are not fully understood. In addition, mitochondrion has not been extensively explored as a therapeutic target for treating RTT. By demonstrating feasibility of performing high-throughput screening of both a small molecule compound library and an siRNA library for hits that rescue the MMP deficit in RTT astrocytes, as well as carrying out further in-depth studies of the identified hits, our study represents a first systematic attempt to address these knowledge gaps.

Compared with other methods to measure MMP, the JC-10 assay is more suitable for high-throughput screening because it is ratiometric in nature. The JC-10 dye is a cationic lipophilic dye that exists as monomers outside of mitochondria and accumulates as J-aggregates inside the mitochondria in live cells. Its J-aggregate form emits fluorescence around the wavelength of 590 nm, while its monomer form emits fluorescence inside the wavelength of 520 nm. The ratio of fluorescence intensity at 590 nm over the fluorescence intensity at 520 nm (F590/520) is sensitive to membrane polarization, serving as a robust and reliable measure of MMP. In this study, JC-10 was exclusively used as a rapid primary readout for our large-scale siRNA and small-molecule library screenings. Candidate hits were subjected to tailored orthogonal validations: isradipine (small molecule) was validated by mitochondrial morphology/ATP assays and *in vivo* mouse experiments, while *LRRC17* (siRNA) was validated by mitochondrial morphology/ATP/calcium activity assays and *in vitro* neuronal morphology using LRRC17 neutralizing antibody and exogenous recombinant LRRC17 protein. These secondary validation assays helped to rule out artifacts specific to the JC-10 assay and maladaptive mitochondrial hyperpolarization, further confirming disease-relevant mitochondrial functional improvement.

In light of our previous report of abnormal Ca^2+^ homeostasis in RTT astrocytes, it is not surprising that several of positive hits identified in our drug screening are known to target Ca^2+^ channels, including cleviprex, isradipine, and lomerizine HCl. Yet it is not clear whether they acted directly through those channels in mediating the rescue of MMP deficit in RTT astrocytes. Nonetheless, we further validated the efficacy of isradipine, a L-type Ca^2+^ channel antagonist, in a series of in *vitro* and in *vivo* assays. It is interesting to observe that isradipine rescued not only the MMP deficit (the original screening criterion), but also other defects of the mitochondria (mitochondrial structure and ATP production) and beyond (spontaneous cytosolic Ca^2+^ activity) in human RTT astrocytes. These broad beneficial effects indicate its therapeutic actions may not be solely mediated by L-type Ca^2+^ channels, with potential off-target or network-level effects possibly also contributing to its efficacy. Moreover, direct administration of isradipine rescued MMP deficit in RTT neurons, suggesting its therapeutic benefit may not be limited to a single cell type when administered in *vivo*. Finally, our data showing chronic isradipine administration provided preliminary evidence of extended lifespan and improved characteristic RTT phenotypes in *Mecp2* knockout mice, serving as the foundation for further preclinical studies as the next step to develop this promising drug candidate. Notably, the neuroprotective effect of L-type calcium channel blockers may be a promising strategy to rescue synaptic dysfunction in RTT models (50). Together, these results suggest this new high-throughput screening platform has the potential to support the discovery of novel targets for developing new treatments for RTT.

Of note, the HTS platform described here is inherently modular and can be readily applied to other pediatric neurodevelopmental disorders by exchanging the patient-derived cellular model, disease-relevant phenotypic readouts, and benchmark controls while preserving the same automated screening and validation pipeline. Importantly, anchoring HTS phenotypes to human disease biology strengthens translational relevance. And the drug repurposing route offers a faster timeline to patients when libraries containing FDA-approved drugs are screened. In parallel, patient-derived peripheral cells offer a feasible translational bridge, as mitochondrial redox imbalance and CoQ10 deficiency have been demonstrated in RTT fibroblasts with mutation-dependent severity and pharmacologic responsiveness (51), further providing candidate biomarkers for patient stratification and target engagement. Together, these approaches enable prioritization of HTS hits with prior pediatric safety, CNS penetrance, and biomarker-linked pharmacodynamics, facilitating rational advancement into early-phase pediatric clinical trials and extension of this platform to related neurodevelopmental disorders.

Compared with the drug screen, our siRNA screen will not only identify novel drug targets but also obtain new insight into the molecular mechanisms underlying the disease, because it will identify candidate genes. In that regard, we are pleased that *TRPC4* is on the list of positive hits, as our previous report linked its overexpression to abnormal Ca^2+^ homeostasis in RTT astrocytes. As for new insights, we identified a secreted protein, LRRC17, a strong candidate mediator whose elevated levels are strongly associated with and likely contribute to mitochondrial deficits in RTT astrocytes, whose knockdown rescued the MMP deficit in RTT astrocytes. *LRRC17* encodes a 37 kDa protein containing five putative LRR domains. It was originally known as p37NB, identified by cDNA subtraction analysis with higher gene expression levels in a S-type neuroblastoma cell line. *LRRC17* has been shown to be involved in bone homeostasis, acting as a negative regulator of RANKL-induced osteoclast differentiation from bone marrow precursors. No reports were found about *LRRC17* working in neurological diseases. Consistent with our finding that siRNA knockdown of *LRRC17* in RTT astrocytes improved MMP, a previous study demonstrated that knocking down *LRRC17* in bone marrow-derived mesenchymal stem cells (BMSCs) promotes mitochondrial quality control by activating mitophagy via mTOR/PI3K inhibition, improving both mitochondrial function and therapeutic outcomes in a mouse model of osteoporosis (52). Through a set of additional experiments, we demonstrated that LRRC17 level is elevated in both RTT astrocytes (carrying different *MECP2* mutations) and medium conditioned by RTT astrocytes using a combination of immunostaining, Western blot, and ELISA; and that the secreted form of LRRC17 mediates the non-cell autonomous impact of RTT astrocytes on neuronal morphology using LRRC17 neutralizing antibody and exogenous recombinant LRRC17 protein. To our knowledge, this is the first time a protein secreted by RTT astrocytes has been shown to impact neuronal dysfunction, which opens the door to a better understanding of neuroglial interaction in RTT pathophysiology.

This study has several limitations that warrant consideration. First, our *in vivo* testing employed only male *Mecp2^−/y^* mice. While this choice is justified by the well-established nature of this model (39), which recapitulates key features of RTT with high penetrance, female *Mecp2*^+/−^ mice are considered to more closely mimic the human condition. Future studies involving female *Mecp2*^+/−^ mice will further demonstrate the relevance of isradipine for clinical development. Second, we characterized mitochondrial function using *in vitro* systems, without performing direct *in vivo* mitochondrial measurements in the intact brain. Thus, the physiological relevance of these mitochondrial changes in living animals remains to be validated. Third, the preclinical data for isradipine were generated from an early-stage, single-center study, and further multi-center validation with larger sample sizes, combined with long-term efficacy and safety assessments, will be required to support translational development. Future studies addressing these limitations will help to strengthen the clinical relevance of our findings.

In summary, our study provided preliminary evidence of a new HTS platform capable of identifying candidate drugs and candidate genes that improve mitochondrial health in RTT astrocytes. We provide the RTT research field with a new tool that not only identifies novel therapeutic drugs and gene targets for treating RTT, but also gains new insights into the mechanisms underlying the disease.

## Data Availability

The raw data supporting the conclusions of this article will be made available by the authors, without undue reservation.

## Glossary

RTT: Rett syndrome
*MECP2*: Methyl-CpG binding protein 2
hESCs: Human embryonic stem cells
iPSCs: Induced pluripotent stem cells
HTS: High-throughput screening
MMP: Mitochondrial membrane potential
FDA: Food and Drug Administration
ELISA: Enzyme-linked immunosorbent assay
*LRRC17*: Leucine rich repeat containing 17
GFP: Green fluorescent protein
WT: Wild type
KO: Knock-out
MT: Mutant
SD: Standard deviation
GFAP: Glial fibrillary acidic protein
AP: Astrocyte progenitors
AR: Aspect ratio
FF: Form factor
RNAi: RNA interference
Pr: Protein
AS: Astrocyte
Ctrl: Control
