# Supplementary material for: A high-throughput screening platform to facilitate treatment development in Rett syndrome
Source: Front Neurol. 2026 May 8;17:1759410. doi: 10.3389/fneur.2026.1759410 (PMC13196345; doi:10.3389/fneur.2026.1759410)
Supplement: Supplementary file 8 [file Supplementary_file_1.pdf]

Supplementary materials

Materials and Methods

NEURON DIFFERENTIATION

Human forebrain neurons were differentiated from hESC/iPSCs as previously described [1]. Briefly, hESC/iPSCs were passaged onto irradiated MEF plates using Dispase (1 U/ml) and cultured in the hESC medium for 5-7 d until 80-90% confluent. For inducing neural differentiation, the neural differentiation medium (NDM) was used for generating neuroepithelial progenitors for 7 days, including DMEM/F12, the Neurobasal medium at 1:1, 1 × N2, 0.5 × B27, 0.5 × Glutamax, and 1 × NEAA (all from Invitrogen), added with 2 µm DMH1 (Torcris Biosciences, catalog: #4126) and 2 µm SB 431542 (Stemgent, catalog: 04-0010-10). The culture medium was changed every day. The NP cells were then dissociated with dispase (1 U/ml) and lifted up gently in the neuronal induction medium (NIM), including DMEM/F12, 1 × N2, and 1 × NEAA for 7 days. To differentiate neurons, NPs were dissociated with TrypLE Enzyme into single cells, filtered and plated on Matrigel-coated plates or on astrocytes in the neuronal differentiation medium, including the Neurobasal medium, 1 × N2, 1 × B27, 0.5 × Glutamax, and 1 × NEAA, with 0.1 µm Compound E (Calbiochem, catalog: #530509), 0.5 µm ROCK inhibitor (Torcris, catalog: #1254).

ASTROCYTE DIFFERENTIATION

Human astrocyte differentiation was performed as previously described with minor modification [2,3]. Briefly, NP media were changed to the astrocyte progenitor medium (APM), including DMEM/F12, 1 × N2, 1 × B27, 1 × NEAA, 10 ng/mL EGF (PeproTech, catalog: 500-P306) and 10 ng/mL FGF2 (Waisman Core). The culture medium was changed every 3d. After 6 months, AP spheres were dissociated with TrypLE Enzyme into single cells and plated on Matrigel-coated plates in the astrocyte medium for terminal differentiation, including DMEM/F12, 1 × N2, 1 × NEAA, 10 ng/ml BMP4, LIF, CNTF (BioSensis, catalog: PE-003-100). Differentiated astrocytes were then fed every 3d and used for experimentation 7d after the start day. Stereological analysis was performed to determine the percentage of GFAP positive cells in each differentiation. Astrocyte cultures with higher than 90% GFAP positive cells were used for subsequent experiments.

MEASUREMENT OF CELLULAR ATP

Levels of cellular ATP were quantified using ATPlite^{TM} Luminescence ATP Detection Assay System (Perkin-Elmer), according to the manufacturer's instructions. Briefly, progenitor cells were seeded into matrigel-coated 96-well plate, differentiated into astrocytes and matured for 7 d. Then, cells were transfected with 1µl siRNA premixed with 0.8µl RNAiMax for 72 h. Cells were lysed and incubated with the luciferase substrate solution. The luminescence was measured using a GloMax

Multi+platereader (Promega). The concentration of ATP was calculated with the calibration curves which were constructed at six calibration standards samples.

MITOCHONDRIAL MORPHOLOGY

Mitochondria were labeled with Rh123 (Thermo Fisher Scientific, catalog # R302) for 30 min at 37 °C. Nuclear were dyed with DAPI for 5 min before imaging. Images were taken using an A1RSi confocal microscope system (Nikon) and 100X objective was used. The form factor (FF) and aspect ratio (AR) of mitochondria was calculated using Image J (RRID:SCR_003070) with mitochondrial morphology plug-in as previously described [4].

IN *VITRO* CALCIUM IMAGING

In vitro calcium imaging was performed as previously described [2]. Briefly, For the hESC/iPSCs derived astrocytes, intracellular Ca^{2+} was indicated by Fluo-4. Cells were bulk-loaded for 15 min at 37˚C in artificial cerebrospinal fluid (aCSF) containing (in mM): 120 NaCl, 3 KCl, 15 HEPES, 1 $MgCl_2$, 2 $CaCl_2$, 20 Glucose (pH7.4), add Fluo-4/AM (12.5 mg/ml), pluronic acid (0.05%), and DMSO (0.1%). After the Ca^{2+} indicator was loaded, cells were transferred to a chamber and Ca^{2+} imaging was performed using a Nikon A1confocal microscope at room temperature. All image data were taken in the frame-scanning mode at 1 frame every 2 seconds. Fluo-4 was excited at 488 nm. The Ca^{2+} imaging data was analyzed using custom-written programs in Python. The metadata and the image data of the raw images were read with python-bioformats. The spontaneous Ca^{2+} elevations from the soma and process of astrocytes were analyzed separately. Ca^{2+}amplitude and frequency were quantified at the single-cell level. Each cell was treated as one independent observation. Importantly, cells were obtained from a minimum of three independent differentiations per group. Differentiations were performed independently on separate days. We have now clarified this in the Methods section to avoid ambiguity.

WESTERN BLOT

Cultured astrocytes from 6-well plate were washed with PBS once and lysed by RIPA lysis buffer, containing protease inhibitor mixture (Roche), sonicated 15 min, incubated 1 h at 4 °C, centrifuged 15 min by 12000rpm at 4 °C, collected cell lysis into Eppendorf tubes, boiled at 95 °C for 5 min before loading into 10% SDS-PAGE gel. Protein concentration was measured using BCA protein assay (Bio-Rad). The samples were separated by SDS-PAGE and then transferred onto nitrocellulose membrane (Whatman). The membrane was blocked by 5% fat-free milk for 1 h at room temperature and then incubated with appropriate primary antibody diluted in 3% BSA solution at 4°C overnight. After incubation with DyLight dye-conjugated secondary antibodies (Thermo Fisher Scientific, catalog #35518 RRID: AB_614942; #SA5–35571 RRID: AB_2556775; dilution1:10,000) for 1 h at room temperature, blots were scanned

by the Odyssey Western Detection system (LI-COR Biosciences), followed by quantification with ImageStudio software (LI-COR Biosciences). For immunodetection, the following antibodies were used for our analysis: rabbit anti-LRRC17 (Novus, catalog # NBP1-83309, RRID: AB_11001463, 1:800; Proteintech, catalog # 20918-1-AP, 1:300); anti-GAPDH (Millipore, catalog # AB2302 RRID: AB_10615768).

IMMUNOSTAINING

The immunostaining experiments were performed as previously described [2,3] with minor modifications. Briefly, cultured cells were fixed with 4% (w/v) PFA in PBS for 10 min at room temperature and washed 3 times with PBS (10 min per wash). For TOMM20-LRRC17 double staining, cells were permeablized with 0.1% Triton X-100 for 5 min, cells were blocked with 1%BSA, 10% normal donkey serum, 0.3M glycine in 0.1% PBS-Tween (blocking buffer) for 1 h at room temperature. For GFAP staining, cells were permeablized with 1% Triton X-100 for 30 min, cells were blocked with 3% normal donkey serum, 0.25% Triton X-100 in PBS (blocking buffer) for 1 h at room temperature. Cells were then incubated with primary antibodies overnight at 4°C, washed 3 times with PBS (10 min per wash), and incubated with the corresponding secondary antibodies for 1 h at room temperature. Primary antibodies included rabbit anti-LRRC17 (Novus, catalog# NBP1-83309, RRID: AB_11001463, 1:400), mouse anti-TOMM20 (Abcam, catalog #ab56783, RRID: AB_945896), anti-GFAP (Millipore, MAB3402 RRID: AB_94844, 1:500; and Dako, Z0334 RRID: AB_10013382, 1:500). Secondary antibodies were conjugated with either AlexaFluor-488 or AlexaFluor-568 (Thermo Fisher Scientific, catalog #A-21206, also A21206 RRID: AB_2535792; Thermo Fisher Scientific, catalog #A10037 RRID: AB_2534013). Nuclei were counterstained with DAPI. Images were taken using an A1RSi con-focal microscope system (Nikon) with 20×, 60× or 100× objectives.

Cited References

1. Chambers SM, Fasano CA, Papapetrou EP, Tomishima M, Sadelain M, Studer L. Highly efficient neural conversion of human ES and iPS cells by dual inhibition of SMAD signaling. Nat Biotechnol. 2009;27(3):275–280. doi:10.1038/nbt.1529
2. Dong QP, Liu Q, Li RH, Wang AX, Bu Q, Wang KH, et al. Mechanism and consequence of abnormal calcium homeostasis in Rett syndrome astrocytes. eLife. 2018;7: e33417. doi:10.7554/eLife.33417
3. Williams EC, Zhong XF, Mohamed A, Li RH, Liu Y, Dong Q, et al. Mutant astrocytes differentiated from Rett syndrome patient-specific iPSCs have adverse effects on wild-type neurons. Hum Mol Genet. 2014;23(11):2968–2980. doi:10.1093/hmg/ddu008
4. Koopman WJH, Visch HJ, Smeitink JAM, Willems PHGM. Simultaneous quantitative measurement and automated analysis of mitochondrial morphology, mass, potential, and motility in living human cells. Nat Pro. 2006; 1(3): 1416–1426. DOI: 10.1038/nprot.2006.171

Supplementary Figure 1 (Fig. S1)

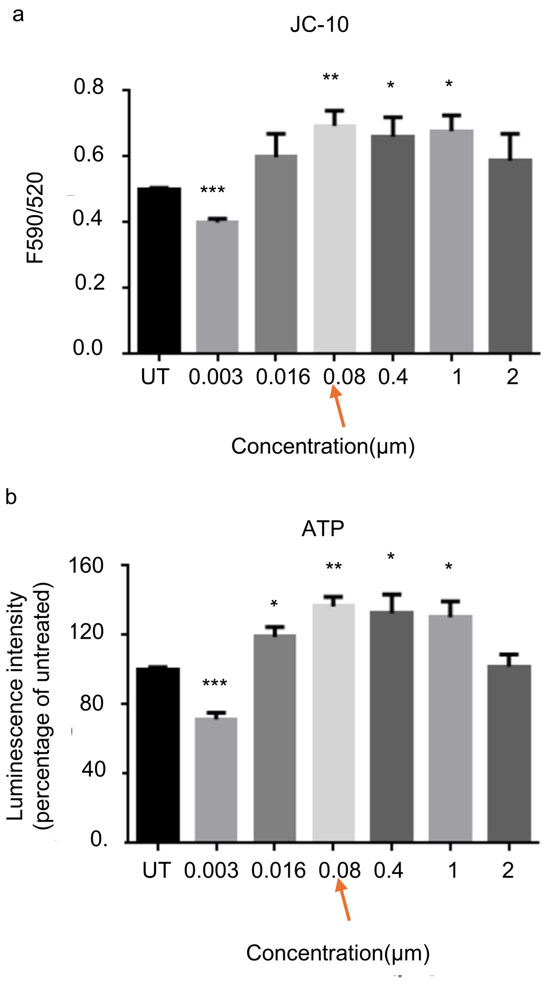

Figure S1

Fig. S1 Dose-response effects of isradipine on mitochondrial membrane potential and cellular ATP levels in V247fs mutant (MT) neurons. a. Concentration-dependent changes in mitochondrial membrane potential measured by JC-10 fluorescence assay.

The 520/590 nm fluorescence ratio was quantified across VCR concentrations ranging from 0.01 to 1.0 μM. A significant dose-dependent increase in the ratio was observed, with an EC_{50} value of 0.08 μM (n=6 independent experiments, mean ± SEM; *$p < 0.05$, **$p < 0.01$, ***$p < 0.001$ vs. vehicle control). b. Dose-response profile of cellular ATP levels following VCR treatment, expressed as a percentage of vehicle-treated control. ATP levels exhibited a concentration-dependent [decline/increase/biphasic change] consistent with mitochondrial functional alterations, with maximal effect observed at 0.08 μM (n=6 independent experiments, mean ± SEM; one-way ANOVA with post hoc test (e.g., Tukey's test), *$p < 0.05$, **$p < 0.01$ vs. vehicle control). Data confirm the dose-dependent bioactivity of VCR on mitochondrial function, supporting its classification as a validated hit from the primary screen,

Supplementary Figure 2 (Fig. S2).

a

b

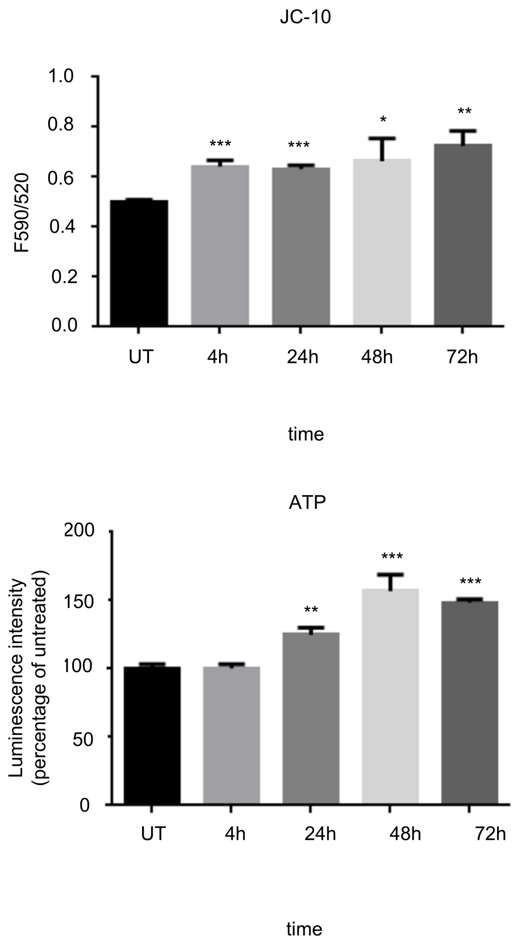

Figure S2

Fig. S2 Time-response effects of isradipine on mitochondrial membrane potential and cellular ATP levels in V247fs mutant (MT) astrocytes. Time-dependent changes in mitochondrial membrane potential measured by JC-10 fluorescence assay. The F590/F520 ratio was quantified at 4, 24, 48, and 72 h post-treatment, and compared to the untreated (UT) control. A significant increase in the ratio was observed as early as 4 h, which was sustained through 72 h (n=6 independent experiments, mean ± SEM; $^{*}p < 0.05$, $^{**}p < 0.01$, $^{***}p < 0.001$ vs. UT). b Time-dependent changes in cellular ATP levels, expressed as a percentage of UT control. ATP levels increased progressively over time, reaching a maximum at 48 h, and were maintained at 72 h (n=6 independent experiments, mean ± SEM; one-way ANOVA with post hoc test (e.g., Tukey's test), $^{*}p < 0.05$, $^{**}p < 0.01$ vs. UT). These data demonstrate that VCR induces a time-

dependent alteration in mitochondrial function, further validating its activity as a confirmed hit.

Supplementary Figure 3 (Fig. S3)

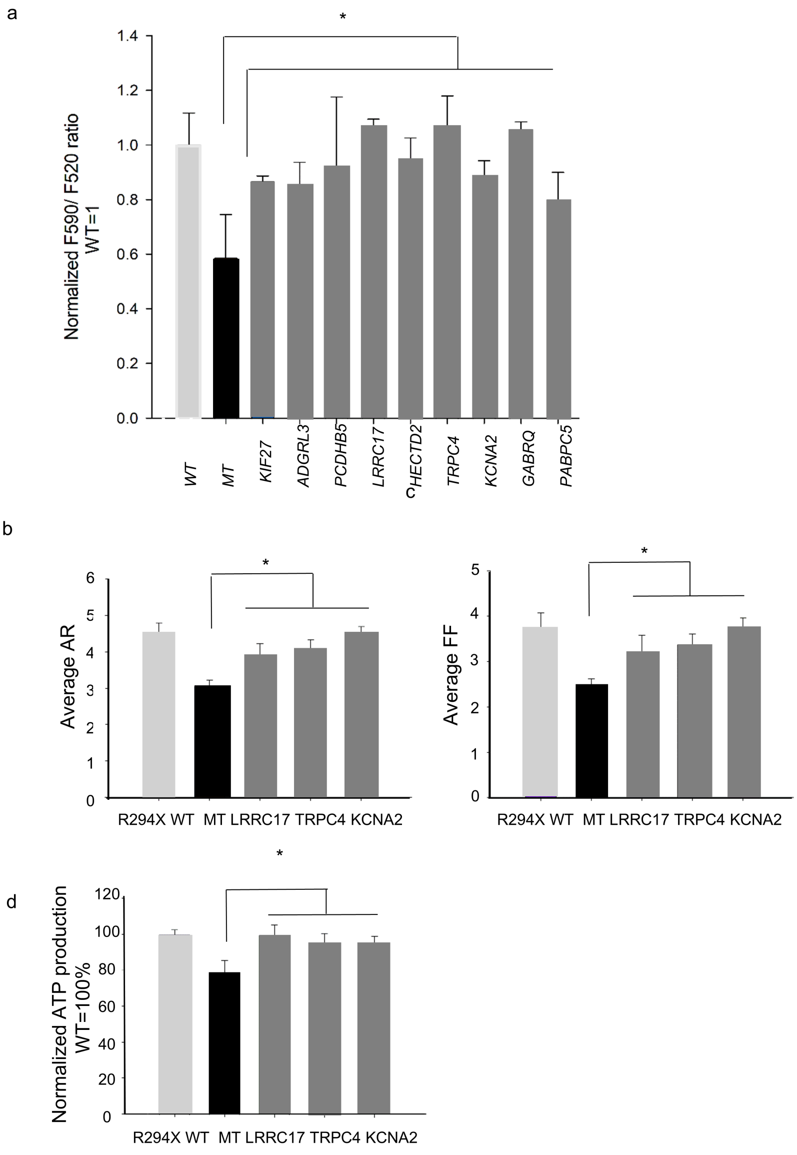

Figure S4

Fig.S3 siRNA screening validation and functional characterization of candidate genes. a. Normalized F590/F520 fluorescence ratio in MT cells transfected with siRNAs targeting the indicated genes, including all primary hits from the initial screening and the R294X WT/MT controls. Data are presented as mean ± SEM. n=6, Blue bars indicate a significant difference (one-way ANOVA with Tukey's post hoc test, $p < 0.05$) compared to the R294X MT control but not the WT control, while magenta bars indicate a significant difference ($p < 0.05$) compared to both. b-d. Functional validation of selected top candidate genes, showing their effects (b, c) mitochondrial morphology, and on (d) cellular ATP levels, confirming their roles in modulating the R294X mutant phenotype.

Supplementary Figure 4 (Fig. S4)

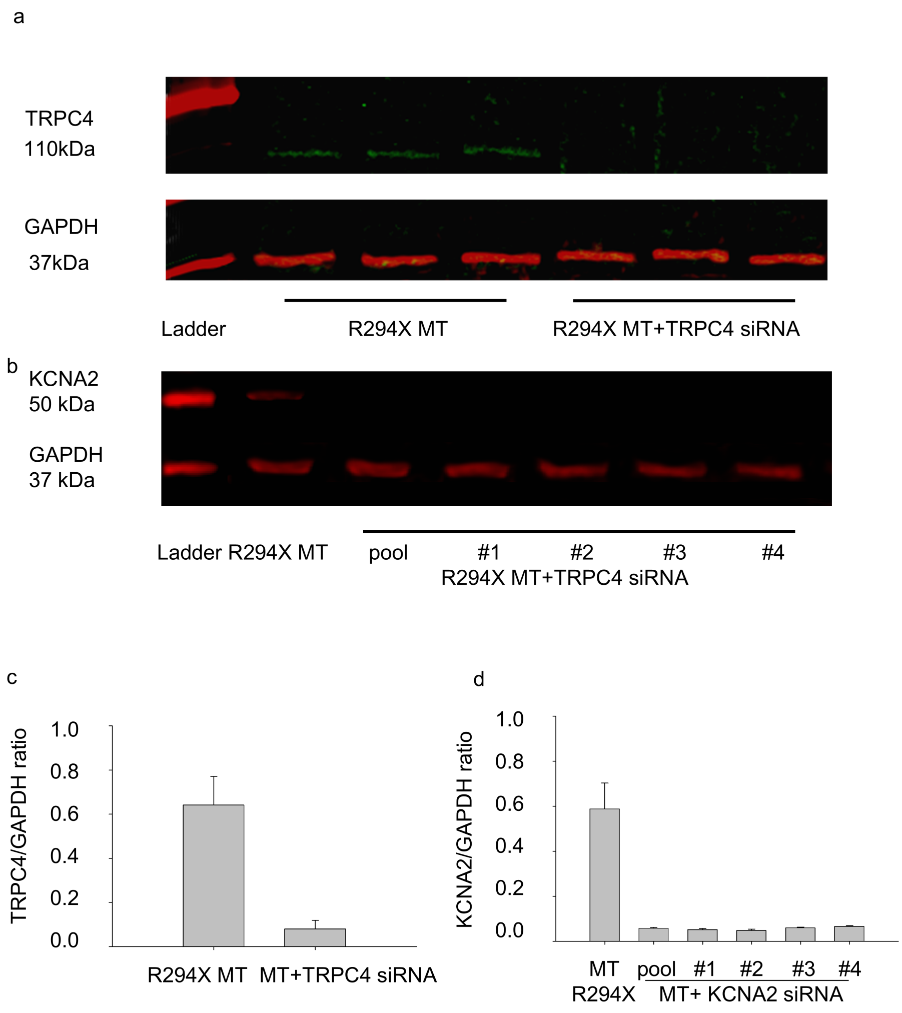

Figure S4

Fig. S4 SiRNA screening hits validation by Western blot. a. Immunoblot analysis shows the expression of TRPC4 protein in R294X MT cells and in R294X MT cells transfected with *TRPC4*-targeting siRNA. GAPDH was used as a loading control. b. Immunoblot analysis shows the expression of KCNA2 protein in R294X MT cells, in a pooled sample of R294X MT cells transfected with *TRPC4* siRNA pool, and in four individual clones (#1- #4) of R294X MT cells transfected with *TRPC4* siRNA. GAPDH was used as a loading control. c. Quantification of the TRPC4/GAPDH ratio demonstrates that

TRPC4 protein expression is efficiently knocked down by *TRPC4* siRNA treatment. d. Quantification of the KCNA2/GAPDH ratio confirms that KCNA2 protein expression is significantly reduced in *KCNA2*-knockdown cells, both in the pool and in individual clones. Data are presented as mean ± SEM (n=6 independent experiments), one-way ANOVA with Tukey’s post hoc test, (****p* < 0.001).

Supplementary Table S1 (Table S1)

Figures/Cell lines	R294X (hiPSCs)	V247fs (hiPSCs)	T158M(hESCs)	KO(hESCs)	H9-GFP(hESCs)
Figure 1	√				
Figure 2a-c			√		
Figure 2e, h			√		
Figure 2d,f,g,i	√	√	√		
Figure 4	√				
Figure 5	√		√	√	
Figure 6	√		√	√	
Figure 7	√				
Figure 8	√				
Figure 9	√ (Astrocyte)				√ (Neuron)
Figure 10					√
Figure S1		√			
Figure S2		√			
Figure S3	√				
Figure S4	√				

Symbols: √ indicates that the listed cell line was used in the corresponding figure panel.

Supplementary Table S2 (Table S2)

Drug Name	Cross BBB	Astrocyte			Neuron			
		JC-10	ATP	mito morph	JC-10	ATP	Ca2+(4h)	mice test
Isradipine (Dynacirc)	YES							
Lomerizine HCl	YES							
Blonanserin (Lonasen)	YES							
Duloxetine HCl (Cymbalta)	YES							
Cleviprex (Clevidipine)	YES							
Edaravone (MCI-186)	YES							
Aripiprazole (Abilify)	YES							
Agomelatine	YES							
Domperidone (Motilium)	YES							
Megestrol Acetate	YES							
Telbivudine (Sebivo, Tyzeka)	YES							
Fluocinolone acetonide (Flucort-N)	YES							
Deferiprone	YES							

Note:
JC10: JC-10 Assay
IF: Immunofluorescence
Mito Morph: Mitochondrial morphology
ATP: ATP detection
CA^{2+}: Calcium activity
Symbols: Green indicates the experimental results were consistent with the original screening data and/or rescued the defects caused by the loss of MECP2 function; Red indicates the experimental results were inconsistent with the original screening data and/or didn’t rescue the defects caused by the loss of MECP2 function; Blank indicates the experiment was not conducted for that drug.

Supplementary Table S3 (Table S3)

Gene Name	NCBI Gene ID	Locus	Dharmacon cat #
PDE6D	5147	chr2:232597146-232645974	M-004310-03
PAX6	5080	chr11:31806339-31908587	M-011098-01
ATP8A1	10396	chr4:42410391-42659122	M-008166-01
AKAP14	158798	chrX:119029935-119054679	M-017696-01
CA8	767	chr8:61101422-61193954	M-009012-01
DCC	1630	chr18:49866541-51062273	M-003880-02
LIPG	9388	chr18:47088426-47119278	M-009601-01
ARID5B	84159	chr10:63661012-63856707	M-026427-01
ATP10B	23120	chr5:159990126-160279219	M-034986-01
GDAP1	54332	chr8:75262617-75279335	M-021225-01
INSM1	3642	chr20:20348764-20351592	M-006535-00
CDO1	1036	chr5:115140429-115152405	M-010336-01
ADCY2	108	chr5:7396342-7830194	M-006798-02
CYP26A1	1592	chr10:94833231-94837641	M-009526-01
CD38	952	chr4:15779930-15850706	M-009222-01
PDE1C	5137	chr7:31792631-32338383	M-007643-01
IL13RA2	3598	chrX:114238537-114252207	M-004598-00
PPARGC1A	10891	chr4:23793643-23891700	M-005111-01
CCND1	595	chr11:69455872-69469242	M-003210-05
GALC	2581	chr14:88399357-88460009	M-011038-01
PVRL3	25945	chr3:110790605-110913016	M-013952-01
NLGN4X	57502	chrX:5808082-6146706	M-010288-00
SMS	6611	chrX:21958690-22012955	M-017273-02
CCNA1	8900	chr13:37005966-37017019	M-003204-02
CAT	847	chr11:34460471-34493607	M-010021-01
DAB1	1601	chr1:57463578-58716211	M-008943-01
PTCH1	5727	chr9:98205263-98279247	M-003924-01
RALGPS2	55103	chr1:178694299-178889237	M-009877-00
RAB8B	51762	chr15:63481727-63559973	M-008744-01
KIF27	55582	chr9:86451614-86536380	M-008723-01
ABCA5	23461	chr17:67144147-67323323	M-004345-00
PLAGL1	5325	chr6:144261436-144385735	M-006546-01
SPATA5L1	79029	chr15:45694518-45713616	M-008899-00
ITGB8	3696	chr7:20370724-20455382	M-008014-02
LRP2	4036	chr2:169983618-170219122	M-012673-01
GATM	2628	chr15:45653321-45672321	M-008900-01
FUT8	2530	chr14:65877309-66210839	M-003668-03
RHOJ	57381	chr14:63671101-63760230	M-010367-01
SLC6A15	55117	chr12:85253266-85306608	M-007373-01
SLC1A3	6507	chr5:36606456-36688436	M-007427-00
NLGN3	54413	chrX:70364680-70391051	M-009084-01
CP	1356	chr3:148847370-148939832	M-009303-01
NDP	4693	chrX:43808023-43832921	M-011091-01
BCL2	596	chr18:60790578-60986613	M-003307-06
HES1	3280	chr3:193853930-193856401	M-007770-01
KAL1	3730	chrX:8496914-8700227	M-011061-01
ABAT	18	chr16:8768443-8878432	M-004060-01
DLL1	28514	chr6:170591293-170599697	M-013302-02
VAT1L	51596	chr16:77822482-78014001	M-008897-00
BMP7	655	chr20:55743808-55841707	M-011592-00
CLEC2D	29121	chr12:9822303-9852151	M-012491-01
SERPINI2	5275	chr3:167159576-167191920	M-012246-01
LIFR	3977	chr5:38475064-38595507	M-008017-01
PCYT1B	9468	chrX:24576203-24690979	M-009611-01
SLC6A1	6529	chr3:11034419-11080935	M-007597-01
SLFN12	91606	chr17:33738080-33759543	M-018142-01
FOXG1	2290	chr14:29236277-29239483	M-019124-00

Gene Name	NCBI Gene ID	Locus	Dharmacon cat #
ASCL1	429	chr12:103351451-103354294	M-008307-01
ID4	3400	chr6:19837600-19842431	M-008913-00
RYBP	23429	chr3:72423743-72495774	M-015936-01
SLC25A18	83733	chr22:18043182-18073647	M-007477-01
ATP1B1	481	chr1:169075946-169337186	M-008381-00
RND3	390	chr2:151324706-151344209	M-007794-02
NR2F2	7026	chr15:96869156-96883492	M-003422-00
GDPD2	26099	chrX:69642880-69653241	M-010666-01
SLC17A6	57084	chr11:22359666-22401046	M-007416-00
HMGCS1	3157	chr5:43287571-43313614	M-009808-01
ARL4A	10124	chr7:12726451-12730558	M-012083-01
SORL1	6653	chr11:121322911-121504471	M-004722-00
TAF9B	51616	chrX:77385244-77395179	M-017217-01
CNTFR	1271	chr9:34551429-34590138	M-007856-00
HIVEP2	3097	chr6:143072603-143266338	M-015324-01
PPIL6	285753	chr6:109711417-109765122	M-009111-01
PDE3A	5139	chr12:20522178-20837041	M-007645-02
APC	324	chr5:112043201-112181936	M-003869-01
PXDNL	137902	chr8:52232136-52722005	M-008450-00
RRAGD	58528	chr6:90074334-90121995	M-016120-01
B3GALT2	8707	chr1:193091087-193223942	M-013692-00
SUPT3H	8464	chr6:44794466-45518819	M-019548-01
INHBA	3624	chr7:41728600-41818976	M-011701-02
ENPP2	5168	chr8:120569316-120651106	M-004601-02
DNAH7	56171	chr2:196602426-196933536	M-009928-01
MTTP	4547	chr4:100485239-100545154	M-008301-01
HS3ST3B1	9953	chr17:14204505-14249492	M-010083-01
SOX3	6658	chrX:139585151-139587225	M-012143-00
FGF14	2259	chr13:102104943-103054124	M-011860-01
ABCC9	10060	chr12:21950323-22089628	M-007307-00
RND2	8153	chr17:41177257-41184058	M-009727-01
KIF1B	23095	chr1:10270763-10441661	M-009317-01
KIF5C	3800	chr2:149632791-149883273	M-019811-01
AHCYL2	23382	chr7:128864854-129070052	M-008588-00
DLX5	1749	chr7:96649701-96654143	M-012031-02
LRRC6	23639	chr8:133584200-133687863	M-018722-01
SLIT2	9353	chr4:20255234-20620788	M-019853-01
MANSC1	54682	chr12:12482217-12503169	M-018215-02
MAP6	4135	chr11:75297962-75379479	M-026713-00
SMOC1	64093	chr14:70346113-70499083	M-013885-01
PLA2G16	11145	chr11:63341943-63381941	M-019760-01
MLF1	4291	chr3:158288952-158324249	M-019478-00
ITSN1	6453	chr21:35014783-35261609	M-008365-01
SEMA5B	54437	chr3:122628039-122747452	M-023584-01
DAAM1	23002	chr14:59655380-59838123	M-012925-00
BOC	91653	chr3:112931374-113160361	M-008413-00
FREM1	158326	chr9:14734663-14910993	M-017026-02
PDGFD	80310	chr11:103777913-104035027	M-008876-01
ZNF528	643123	chr19:52901120-52921657	M-014891-01
RSPH4A	345895	chr6:116937641-116954148	M-032884-00
LRP4	4038	chr11:46867961-46940173	M-027194-01
PABPC5	140886	chrX:90689596-90693583	M-015277-00
ZNF501	220082	chr3:44771097-44778575	M-007118-01
KLHL15	28942	chrX:24001832-24045303	M-024931-01
LRRIQ1	84128	chr12:85430098-85638883	M-032523-01
C6orf165	221927	chr6:88117689-88174191	M-018380-01
SALL2	6297	chr14:21989231-22005337	M-027199-01

Gene Name	NCBI Gene ID	Locus	Dharmacon cat #
WNT3	7473	chr17:44841686-44896082	M-009712-00
CNTN3	5067	chr3:74311721-74570343	M-007857-01
PCDH8	5100	chr13:53418108-53422775	M-011349-00
IRS1	3667	chr2:227596032-227663506	M-003015-01
ARMC2	84060	chr6:109169618-109295352	M-018191-01
ZNF521	25925	chr18:22641887-22932214	M-013957-00
NTN4	59277	chr12:96043030-96184536	M-031987-01
LRFN5	145581	chr14:42076763-42373752	M-018762-00
GPC4	2239	chrX:132435063-132549205	M-011271-00
SEMA3D	223117	chr7:84624871-84751247	M-016982-01
KIAA1407	57515	chr3:113682983-113775460	M-014068-00
TOX	9760	chr8:59717976-60031767	M-020339-01
GNG3	2785	chr11:62457733-62494857	M-012804-02
CARD8	22900	chr19:48711342-48761450	M-004454-00
PCDHB5	56131	chr5:140514799-140517704	M-013524-00
STMN4	81551	chr8:27093813-27115903	M-016810-00
IGFBPL1	347252	chr9:38406524-38424444	M-026140-02
ST18	9705	chr8:53023391-53322439	M-008451-00
PCDHB11	56138	chr5:140579347-140582618	M-013527-00
MOB3B	64839	chr9:27325206-27529850	M-018160-00
ZNF572	126820	chr8:125985538-125991630	M-007133-00
ENKUR	54875	chr10:25270907-25351208	M-015999-01
CRISPLD1	83686	chr8:75896707-75946793	M-016681-01
PCDHGA10	56151	chr5:140710251-140892548	M-013294-00
COL3A1	1281	chr2:189839098-189877472	M-011012-01
SALL4	57167	chr20:50400582-50419048	M-007033-01
COL24A1	255631	chr1:86194915-86622121	M-018815-01
LRRC17	10234	chr7:102453307-102715288	M-020025-02
SPON1	10418	chr11:13984183-14289679	M-012730-01
PTX3	5806	chr3:156977531-157221415	M-017765-00
NELL2	4753	chr12:44902057-45307711	M-012185-00
NAP1L3	4675	chrX:92925924-92928682	M-011879-01
IRAK1BP1	11213	chr6:79577188-79608320	M-024865-01
DPPA4	55211	chr3:109044987-109056419	M-020766-01
PCDHB8	56134	chr5:140557370-140560081	M-013280-00
ZBBX	79783	chr3:166958076-167098085	M-014412-01
NEBL	10529	chr10:21068902-21463852	M-020114-00
PCDHB4	56130	chr5:140501580-140505201	M-013281-00
SNCA	6622	chr4:90645249-90763142	M-011109-00
DOK5	55816	chr20:53092265-53267710	M-019050-01
FAM65B	84904	chr6:24804512-24911195	M-013598-00
PCDHB13	56140	chr5:140593508-140596993	M-013529-00
C6orf118	400120	chr6:165693152-165723111	M-021302-01
EFNB1	1947	chrX:68048839-68062006	M-003658-01
CALB1	793	chr8:91070837-91095107	M-011989-02
FSIP1	51266	chr15:39892231-40075039	M-018333-01
ARRDC4	84617	chr15:98503932-98517068	M-019366-01
TMEM47	83604	chrX:34645180-34675405	M-021352-01
ZNF562	147929	chr19:9759337-9785776	M-020609-01
SETBP1	26040	chr18:42260137-42648475	M-013930-00
ZNF248	56951	chr10:38065453-38147012	M-014069-00
SOSTDC1	25928	chr7:16501105-16505474	M-013959-02
PCDHB16	56143	chr5:140560979-140565796	M-013156-00
SEPP1	6414	chr5:42756919-42812024	M-012062-01
MYL12A	103910	chr18:3247527-3256234	M-021498-01
SRGAP1	57522	chr12:64238540-64541613	M-026974-00
SNN	8303	chr16:11762288-11836648	M-018536-01

Gene Name	NCBI Gene ID	Locus	Dharmacon cat #
NHLH1	4808	chr1:160336860-160342638	M-015321-01
ZNF300	7702	chr5:150273953-150284545	M-015144-00
SYT4	6860	chr18:40847856-40857615	M-014054-00
NEFL	4747	chr8:24808468-24814383	M-020082-01
CELF2	10659	chr10:11047258-11378672	M-012741-01
SDK1	221935	chr7:3341079-4308631	M-018964-01
ZNF558	339327	chr19:8920381-8933565	M-015741-00
CNKSR2	22866	chrX:21392535-21672813	M-020433-00
UNC13B	10497	chr9:35161988-35405332	M-012268-01
ZNF229	7779	chr19:44930425-44952665	M-023741-01
CRB1	23418	chr1:197170591-197447585	M-012404-01
ATOH1	474	chr4:94750077-94751142	M-008915-00
DCX	1641	chrX:110537006-110664835	M-011113-00
ZNF354C	79766	chr5:178487606-178507691	M-014199-01
BTBD11	25894	chr12:107712196-108053419	M-015774-01
APLP1	333	chr19:36359400-36370699	M-004178-01
ZNF132	7691	chr19:58944180-58951589	M-019560-01
ZMAT4	79698	chr8:40388110-40755343	M-014398-01
CDH20	1250	chr18:59157774-59222365	M-013506-00
ZNF560	339327	chr19:9577030-9609279	M-016202-01
NHS	4810	chrX:17393542-17754113	M-032018-00
ROBO2	6092	chr3:77089293-77699114	M-023273-01
PLXDC2	84898	chr10:20105371-20569115	M-015203-00
PACRG	135138	chr6:161768589-163745505	M-016948-01
GRB14	2888	chr2:165349322-165478360	M-019810-01
PREX1	57580	chr20:47240792-47444420	M-010063-01
PCDHB6	56132	chr5:140529838-140532868	M-013525-00
MN1	4330	chr22:28144264-28197486	M-011336-01
CXXC4	80319	chr4:105389462-105416058	M-008812-00
FSIP2	401024	chr2:186603354-186698016	M-018151-00
SLITRK3	22865	chr3:164904507-164914469	M-020435-00
MMRN1	22915	chr4:90816051-90875780	M-012390-00
ZNF454	100132874	chr5:178368193-178393218	M-018860-01
PCDHGB5	56127	chr5:140710251-140892548	M-013289-00
PROM1	8842	chr4:15969848-16085623	M-010630-01
PSMD5	5711	chr9:123578331-123605299	M-012006-00
PCDH17	27253	chr13:58205788-58303065	M-012528-02
GNAO1	2774	chr16:56225250-56391356	M-009486-02
PCDHB12	56139	chr5:140588290-140591698	M-013528-01
KHDRBS2	202559	chr6:62389864-62996100	M-016897-00
PCDHB3	56129	chr5:140480233-140483406	M-013282-00
SEMA5A	9037	chr5:9035137-9546233	M-019490-01
RGS1	5996	chr1:192544856-192549159	M-009501-03
WDR44	11040	chrX:117480035-117583923	M-018913-00
PCDHB15	56142	chr5:140625146-140627801	M-013531-00
SNTG1	54212	chr8:50824596-51705427	M-021231-01
VANGL2	57216	chr1:160370363-160398468	M-010581-01
PCDHB14	56141	chr5:140603077-140605860	M-013530-00
ELAVL3	1996	chr19:11562142-11591803	M-011264-00
LHX9	56956	chr1:197881634-197899273	M-031943-00
SCGN	10505	chr6:25652428-25702008	M-013631-01
HHIP	64399	chr4:145564067-145659881	M-013018-00
NBEA	26960	chr13:35516423-36705514	M-015419-01
GNG11	2791	chr7:93551015-93555826	M-016036-01
OSBPL11	114881	chr3:125247701-125314381	M-008415-00
SPA17	53340	chr11:124492741-124564687	M-007677-02
SKIDA1	389856	chr10:21802408-21814611	M-032220-00

Gene Name	NCBI Gene ID	Locus	Dharmacon cat #
CASC1	23395	chr12:25205180-25348094	M-027150-01
SIPA1L2	57569	chr1:232533711-232651243	M-021865-01
ZIC3	7547	chrX:136648345-136654259	M-011799-00
LAMA2	3908	chr6:129204285-129837710	M-011070-01
ZNF350	59348	chr19:52467592-52490079	M-017814-01
PCDH18	54510	chr4:138440073-138453629	M-013372-01
LRRTM4	80059	chr2:76974849-77749502	M-018334-01
VCAM1	7412	chr1:101185195-101204601	M-013351-01
SHC3	53358	chr9:91620685-91793682	M-017022-01
ARMC3	84687	chr10:23216953-23326514	M-017744-01
EPB41L4A	64097	chr5:111498314-111755010	M-021335-01
DNAI1	27019	chr9:34458810-34520982	M-021414-00
DNALI1	7802	chr1:38022519-38061586	M-021414-00
COBLL1	22837	chr2:165541257-165697928	M-020477-01
SEMA3C	10512	chr7:80371853-80548667	M-007757-01
PLEKHA5	54477	chr12:19282625-19529333	M-015385-00
VGLL3	389136	chr3:86987122-87040257	M-031975-02
NKAIN3	79582	chr8:63161500-63903628	M-018052-01
GRAMD1C	653689	chr3:113557680-113666021	M-020545-01
FAM107A	11170	chr3:58549844-58563491	M-012785-01
THSD4	79875	chr15:71433787-72075722	M-014463-01
WDR16	146845	chr17:9479943-9546776	M-018465-00
ZFP14	81029	chr19:36825354-36870105	M-031093-01
SORBS2	8470	chr4:186506597-186877870	M-011479-01
EFHC2	80258	chrX:44007127-44202923	M-018562-01
FAM81B	123872	chr5:94727047-94786144	M-015447-01
TOX3	27324	chr16:52471917-52581714	M-022555-01
SRGAP3	9901	chr3:9022275-9291369	M-014175-01
INTU	55620	chr4:128554086-128637934	M-031873-00
NRARP	11103	chr9:140194082-140196703	M-032409-00
DENND1B	163486	chr1:197473878-197744623	M-016923-02
CHMP2A	27243	chr19:59062932-59066486	M-020247-00
IL33	90865	chr9:6215785-6257983	M-015122-01
HEPACAM	220296	chr11:124789145-124806308	M-015466-01
C1orf192	54995	chr1:161284165-161337673	M-022350-01
ALG13	79868	chrX:110924345-111003875	M-014459-01
SPOCK1	6695	chr5:136310986-136835018	M-013724-01
ZMAT1	79091	chrX:101137259-101187039	M-024776-01
LRRC55	374659	chr11:56949220-56959188	M-026648-01
STOX2	219736	chr4:184826508-184938875	M-024110-01
CADM2	253559	chr3:85008132-86123579	M-016035-01
ZFP3	124961	chr17:4981753-4999669	M-016831-01
WLS	79971	chr1:68167148-68698284	M-018728-01
TMTC4	79833	chr13:101256089-101327103	M-015004-01
WDR49	151176	chr3:167196472-167371289	M-019275-01
CCDC164	401672	chr2:26624783-26679579	M-016558-00
ZNF676	79922	chr19:22361902-22379753	M-032305-01
SLC44A5	140919	chr1:75667815-76076799	M-016900-01
DMXL2	23312	chr15:51739920-51914967	M-014049-01
ZNF474	80228	chr5:121465214-121489266	M-032031-00
MCTP1	79772	chr5:94042288-94620279	M-016557-01
CNR1	1268	chr6:88849584-88875767	M-004711-02
LPAR4	2846	chrX:78003205-78012578	M-005558-01
LPHN3	23266	chr4:62362838-62938168	M-005652-02
GPR98	84059	chr5:89854616-90460033	M-005656-02
S1PR1	1901	chr1:101702304-101707076	M-003655-02
CXCR4	7852	chr2:136871918-136875725	M-005139-02

Gene Name	NCBI Gene ID	Locus	Dharmacon cat #
SORCS1	114815	chr10:108333420-108924466	M-013422-01
S1PR3	1903	chr9:91605777-91620069	M-005208-02
OMG	4974	chr17:29421944-29704695	M-010106-01
LPAR1	1902	chr9:113636053-113800365	M-003656-02
TRPC4	7223	chr13:38210772-38443939	M-006510-02
GABRG2	2566	chr5:161494647-161582545	M-006174-03
CNGA3	1261	chr2:98962617-99015064	M-006159-02
SCN1A	6323	chr2:166845669-167005642	M-006297-02
KCNN3	3782	chr1:154669941-154842754	M-006270-02
GABRQ	2569	chrX:151806636-151821825	M-006177-00
GRIA3	2892	chrX:122318095-122624766	M-006186-02
KCNK10	54207	chr14:88646451-88793256	M-006255-00
KCNA2	3737	chr1:111136201-111174096	M-006212-00
GRIK3	2899	chr1:37261127-37499844	M-006191-02
CACNA2D1	781	chr7:81579417-82073031	M-020206-01
EPHA7	2045	chr6:93949739-94129300	M-003119-02
JAK2	3717	chr9:4985244-5128183	M-003146-02
TGFBR1	7046	chr9:101867411-101916473	M-003929-02
KCNH8	3777	chr3:19190016-19577135	M-006238-01
PAK1	5058	chr11:77033059-77185108	M-003521-04
MAP2K6	5608	chr17:67410837-67538470	M-003967-01
FGFBP3	1212	chr10:93666344-93669258	M-003132-04
FGFR2	2263	chr10:123237843-123357972	M-003132-04
EFNA5	1946	chr5:106712589-107006596	M-011649-01
NTRK2	4915	chr9:87283465-87638505	M-003160-02
CASK	8573	chrX:41374188-41782287	M-005311-01
NUAK2	81788	chr1:205271190-205290883	M-005374-01
NUAK1	9891	chr12:106457124-106533811	M-004931-00
ADRBK2	157	chr22:25960860-26125258	M-004326-02
PDGFRA	5156	chr4:55095263-55164412	M-003162-04
ERBB4	2066	chr2:212240441-213403352	M-003128-03
AK5	26289	chr1:77747661-78025654	M-004897-02
RPS6KA6	27330	chrX:83313353-83442943	M-004670-01
MAPK10	5602	chr4:86936275-87374283	M-004324-00
EFNB3	1949	chr17:7608519-7614693	M-011261-00
EPHA4	2043	chr2:222282746-222437010	M-003118-02
PPAP2B	8613	chr1:56960418-57045257	M-017312-01
PPP2R2B	5520	chr5:145969066-146461083	M-003022-02
PTPRZ1	5803	chr7:121513158-121702090	M-009685-01
DUSP16	80824	chr12:12626215-12715448	M-007890-00
ADAMTSL1	92949	chr9:18474078-18910947	M-013437-01
ADAMTS3	9508	chr4:73146685-73434516	M-005773-00
CPE	1363	chr4:166300096-166419482	M-005823-00
SENP7	57337	chr3:101043117-101232085	M-006035-01
PAPPA	5069	chr9:118916070-119164600	M-005130-02
HECTD2	57167	chr10:93066718-93371217	M-007198-00
MID1	4281	chrX:10413349-10851809	M-006537-01
FSD1L	84971	chr9:108210314-108311385	M-014752-03
RNF144B	25822	chr6:18387580-18469105	M-025119-01
PJA1	64219	chrX:68380580-68385365	M-007045-01
PCGF5	84333	chr10:92922768-93044088	M-007089-01

Supplementary Table S4 (Table S4)

**Cell type**	**Astrocyte**							**Neuron**
**Gene Name**	**JC-10**	**WB**	**IF**	**Mito morph**	**ATP**	**Neuron morph**	**CA2+**	**IF**
LRRc17								
KIF27								
TRPC4								
KCNA2								
ADGRL3								
PABPC5								
HECTD2								
GABRQ								
PCDHB5								

Note:
JC10: JC-10 Assay
IF: Immunofluorescence
Mito Morph: Mitochondrial morphology
ATP: ATP detection
CA^{2+}: Calcium activity
Symbols: Green indicates the experiment was performed and knocking down that candidate gene rescued the defects caused by the loss of MECP2 function; Blank indicates the experiment was not conducted.
